# Spatio-temporal ecology of sympatric felids on Borneo. Evidence for resource partitioning?

**DOI:** 10.1371/journal.pone.0200828

**Published:** 2018-07-20

**Authors:** Andrew J. Hearn, Samuel A. Cushman, Joanna Ross, Benoit Goossens, Luke T. B. Hunter, David W. Macdonald

**Affiliations:** 1 Wildlife Conservation Research Unit (WildCRU), Department of Zoology, University of Oxford, Oxford, United Kingdom; 2 US Forest Service, Rocky Mountain Research Station, Flagstaff, Arizona, United States of America; 3 Danau Girang Field Centre, c/o Sabah Wildlife Department, Wisma Muis, Kota Kinabalu, Sabah, Malaysia; 4 Sabah Wildlife Department, Wisma Muis, Kota Kinabalu, Sabah, Malaysia; 5 Organisms and Environment Division, Cardiff School of Biosciences, Cardiff University, Sir Martin Evans Building, Museum Avenue, Cardiff, United Kingdom; 6 Sustainable Places Research Institute, Cardiff University, Cardiff, United Kingdom; 7 Panthera, New York, NY, United States of America; Universita degli Studi di Napoli Federico II, ITALY

## Abstract

Niche differentiation, the partitioning of resources along one or more axes of a species’ niche hyper-volume, is widely recognised as an important mechanism for sympatric species to reduce interspecific competition and predation risk, and thus facilitate co-existence. Resource partitioning may be facilitated by behavioural differentiation along three main niche dimensions: habitat, food and time. In this study, we investigate the extent to which these mechanisms can explain the coexistence of an assemblage of five sympatric felids in Borneo. Using multi-scale logistic regression, we show that Bornean felids exhibit differences in both their broad and fine-scale habitat use. We calculate temporal activity patterns and overlap between these species, and present evidence for temporal separation within this felid guild. Lastly, we conducted an all-subsets logistic regression to predict the occurrence of each felid species as a function of the co-occurrence of a large number of other species and showed that Bornean felids co-occurred with a range of other species, some of which could be candidate prey. Our study reveals apparent resource partitioning within the Bornean felid assemblage, operating along all three niche dimension axes. These results provide new insights into the ecology of these species and the broader community in which they live and also provide important information for conservation planning for this guild of predators.

## Introduction

Niche differentiation has long been recognised as an important mechanism whereby ecologically similar, sympatric species may reduce both exploitative and interference interspecific competition, and thus enhance coexistence. Such resource partitioning may be facilitated by the evolutionary displacement of morphological characters [1]) as well as via behavioural mechanisms, and is thought to operate primarily along three main axes of the niche dimension: habitat, food and time [2,3].

The extant members of the *Felidae* share a remarkably conserved morphology and as obligate carnivores, typically specialising in mammalian prey, they likely experience significant intra-guild competitive forces. As such, sympatric guilds of felids provide a useful focal group in which to explore hypotheses pertaining to mechanisms of co-existence. Among felids, several authors have provided evidence of behavioural mechanisms which may facilitate co-existence, including differential use of space [4], prey species and size classes [5] and temporal segregation [6], or a combination of these mechanisms [7]. As with many ecological relationships, body size and morphological similarity are thought to be key factors influencing the competitive interactions among felids. Felids of comparable size are more likely to take more similar prey and thus interspecific competition should be highest among felid pairs as they become more closely matched in size [8].

The forests of Borneo support an assemblage of five felids, including the Sunda clouded leopard (*Neofelis diardi*), bay cat (*Catopuma badia*)–a Bornean endemic–, marbled cat (*Pardofelis marmorata*), leopard cat (*Prionailurus bengalensis*) and flat-headed cat (*Prionailurus planiceps*). Understanding the mechanisms which facilitate co-existence within this threatened wild felid guild may inform conservation strategies; yet to date there is limited understanding of the ecology of any of these species, and very little information about resource partitioning.

The flat-headed cat has morphological adaptations for hunting prey in shallow water, and incidental observations [9] and presence-only habitat suitability modelling [10,11] suggest that this felid is restricted to low lying, wetland forest habitats not heavily utilized by the other guild members. Uniquely among the Bornean felids, leopard cats appear to have an affinity for disturbed forest habitats and oil palm plantations where they seek their primary prey, murid rodents [12,13]. Presence-only ecological niche modelling of leopard cats on Borneo confirms these associations [14], while similar predictive models of Sunda clouded leopard, marbled cat and bay cat distributions [15–17] suggest these felids select forest habitats and avoid oil palm plantations. However, while Sunda clouded leopards and marbled cats are regularly recorded walking along the ground [18,19], morphological adaptations for an arboreal lifestyle [20] and incidental observations of them hunting arboreal prey [21,22] suggest at least partial habitat segregation from that of the other, presumably terrestrial, guild members.

Fine-scale differences in habitat use may also explain co-existence within this felid assemblage. Wearn et al. (2013) [23] showed that detection probabilities in a highly degraded Bornean forest were significantly higher for clouded leopards along logging roads and skid trails, and higher for marbled cats along skid trails only, whereas no such associations were found for bay cats or leopard cats. Conversely, Mohamed et al. (2013) [24] showed that leopard cat encounter rates from off-road camera traps were only 3.6–9.1% of those for on-road traps.

Bornean felids may also partition along spatial axes by responding to habitat variables at different spatial scales. Several studies that have assessed multi-scale habitat selection optimization have shown that animals often respond most strongly to human disturbance at relatively coarse scales, often far exceeding that of the animal’s home range, while typically selecting habitat variables for foraging or resting at finer spatial scales [25,26].

Telemetry data and camera trap records obtained throughout the diel period show that Sunda clouded leopards and leopard cats are primarily nocturnal [12,27,28]. Camera trap records have been used to describe activity patterns of the other three felids, but very small sample sizes have prevented robust inference [29]. It is possible, however, that Bornean felids partition along the temporal axis.

In the absence of further detailed ecological information, examination of body sizes may provide insights into the potential competitive interactions within the Bornean felid assemblage. The Sunda clouded leopard appears to exhibit a large degree of sexual dimorphism, with males reaching weights of around 24 kg whereas females are around 12 kg. The four other members of the felid guild are significantly smaller than this, and thus presumably competitively subordinate, and exhibit largely overlapping body sizes (Table 1). Accordingly, competition for prey is likely to be highest among the four smaller species.

**Table 1:** Body weight ratios of adult, free-ranging Bornean felids.

Species: Mean weight (SE) in kg, sample size	Sunda clouded leopard		bay cat	marbled cat	leopard cat	flat-headed cat
	males	females				
Sunda clouded leopard males: 24.4 (0.8), n = 5 [27,30]	1	2.0	8.7	10.2	11.6	12.8
Sunda clouded leopard females: 12.5 (3.2), n = 4 [27,31]		1	4.5	5.2	6.0	6.6
bay cat: 2.8 (0.6), n = 3 [32,33]			1	**1.2**	**1.3**	**1.5**
marbled cat: 2.4 (0), n = 2 [20,21]				1	**1.1**	**1.3**
leopard cat: 2.1 (0.4), n = 19 [34,35]					1	**1.1**
flat-headed cat: 1.9 (0.3), n = 4 [20]						1

Body weight ratios (heavier/lighter) in bold highlight species pairs with very similar body sizes (ratio <2), which are predicted to compete for similar prey [8].

In this study, we draw together an extensive dataset on Bornean felids derived from camera trap surveys of multiple areas in Sabah, Malaysian Borneo, to examine resource partitioning and identify potential mechanisms of co-existence within the Bornean felid assemblage. We use the following approaches: (1) Multi-scale habitat modelling to identify the habitat variables and scales that influence Bornean felid occurrence across the landscape and to refine current predictions of these felids’ distributions; (2) All-subsets logistical regression to explore the spatial co-occurrence of Bornean felids with other Bornean mammals and birds, to identify potential candidate prey species; (3) Modelling of temporal activity patterns to assess and compare temporal activity patterns within the felid guild and to quantify overlaps in temporal activity between felids and potential candidate prey. We hypothesise that the highest levels of segregation along the spatial, temporal, and prey niche dimensions will be exhibited by species pairs with the greatest overlap in body size (i.e., between the 4 smaller species). We hypothesise that leopard cats will select disturbed areas at fine spatial scales and that, conversely, the Sunda clouded leopard, bay cat, marbled cat and flat-headed cat will all exhibit broad scale avoidance of disturbed habitats, but will vary in their selection of optimal foraging habitat at fine scales. We also hypothesise that species that are predicted to experience a higher rate of intra-guild killing (i.e., all four small felids) will exhibit spatio-temporal avoidance of the larger and competitively dominant Sunda clouded leopards. Lastly, we predict that female Sunda clouded leopards, which will often be accompanied by cubs, will attempt to avoid encounters with male Sunda clouded leopards.

## Material and methods

### Ethics statement

The Economic Planning Unit of Malaysia, Sabah Biodiversity Council, Sabah Parks, Sabah Forestry Department, Sabah Wildlife Department and Yayasan Sabah reviewed all sampling procedures and approved permits for the work conducted. We applied non-invasive methods for data gathering and hence approval from an Institutional Animal Care and Use Committee or equivalent animal ethics committee was not required.

### Data collection

**Fig 1:**
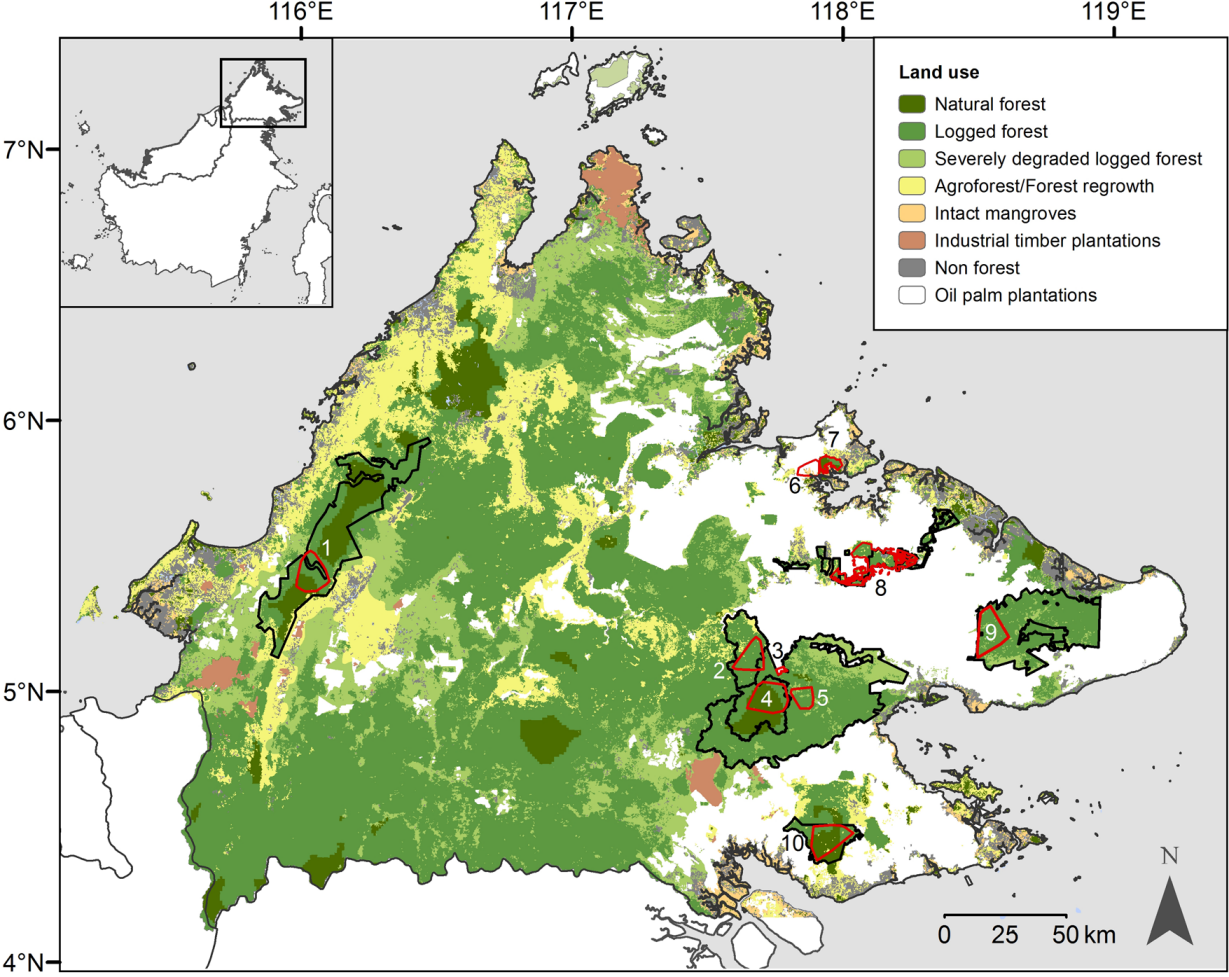
The locations of the eight forest and two oil palm plantation study areas in Sabah, Malaysian Borneo. Numbered polygons represent the different study areas, defined by the locations of the outermost camera stations: 1. Crocker Range Park; 2. Malua Forest Reserve; 3. Danum Palm Plantation; 4. Danum Valley Conservation Area (surveyed on two separate occasions); 5. Ulu Segama Forest Reserve; 6. Minat Teguh plantation; 7. Kabili-Sepilok Forest Reserve; 8. Lower Kinabatangan Wildlife Sanctuary; 9. Tabin Wildlife Reserve; 10. Tawau Hills Park (surveyed on two separate occasions). Inset shows the island of Borneo. Land use data from 2010 [36].

Between May 2007 and December 2014, we conducted intensive, systematic camera trap surveys of eight forest areas and two oil palm plantations in Sabah, Malaysian Borneo (Fig 1). Study areas provided a broadly representative sample of the spectrum of land uses, elevations, anthropogenic disturbance and forest fragmentation present in the state. We deployed cameras at 578 camera stations over an elevation range of 5–1442 m, and accumulated a total of 72,490 trap days (S1 and S2 Tables). For further details of the survey approach, see [18]. For all analyses, we reduced the number of camera trap detections for each species to one detection per hour, per camera station.

### Multi-scale habitat modelling

We selected a suite of habitat variables (S3 Table) we believed may be strongly related to Bornean felid occurrence based on previous research [11,14–18,37]. We used a 250 m spatial resolution 2010 land cover map [38] and a 50 m resolution 2010 forest quality and land use cover map of Borneo [36]. We used an unpublished historic land cover layer, developed by the Sabah Forestry Department (SFD), which provides an estimation of forest type cover (24 classes) based on soil and elevation associations at a point in time before anthropogenic modification. We clipped this layer to the Gaveau et al. (2014) [38] 2010 forest cover extent, to account for forest conversion. We used a 30 m resolution canopy cover layer [39] and a 30m resolution digital elevation model [40], from which we derived topographical roughness using the Geomorphometry & Gradient Metrics Toolbox in ArcGIS 10.2.2 (Environmental Systems Research Incorporated, ESRI, Redlands, CA, USA, 2011), and a model of human footprint [41].

We developed a multiscale model of each felid species’ occurrence as a function of environmental predictor variables in three stages, following the recommendations of McGarigal et al. (2016) [42]. First, we conducted univariate scaling with logistic regression to identify the spatial scale at which each habitat variable was most strongly related to each Bornean felid’s occurrence (e.g., [25,43]), using total number of detections as the response variable, and applying the following spatial scales of analysis: 120, 240, 480, 960, 1920, 3840 and 7680 m focal-radius moving window. We selected the best-supported scale for each variable based on lowest Akaike’s Information Criterion (AIC; [44]). Due to the clustered nature of the camera trap stations within study areas, a Mixed model would have been preferable, but using this approach the models failed to converge and so we used a standard Generalised Linear model [45].

We reduced the number of variables retained for the final model in two steps. First, we removed all variables for which *p* > 0.2 (e.g., [46]). Second, we assessed multi-collinearity among all possible pairs of scale-optimised variables to identify variables with a Pearson’s correlation value > 0.7, and we retained the variable in each such pair with the lower AIC value. Lastly, we ran all-subsets logistic regression analyses with Dredge function in the MuMIn R package (version 3.4.3; R Development Core Team, 2017 [47] to obtain final model averaged coefficients for each species. We created maps of probability of occurrence across Sabah as a function of the final averaged model for each felid using the equation p = e^z^ / (1 + exp(z)), where z is the linear combination of coefficients multiplied by the independent variables. This modelling approach does not account for the variance in detection probability between camera stations. However, given that there are violations of both closure and independence in our data set, we chose not to use an occupancy approach. It is not necessary to use an occupancy modelling approach to obtain useful inferences about habitat selection. For example, the overall measure of strength and direction of the results are unaffected by imperfect detection [48]. Kelly (2008) [49] also found that unadjusted capture frequencies were highly correlated with adjusted abundance using data from Tobler et al. (2008) [50]. Additionally, Banks-Leite et al. (2013) [49] found that adjusted abundance estimates entail much higher data requirements including repeat analysis in each location, which was not part of the sampling design for this study.

We also used univariate logistic analyses to explore the relationship between Bornean felid occurrence and two fine-scale variables that are not available at the regional extent, including whether the cameras were placed on unsealed logging roads and topographical ridgelines. Previous studies have shown significant relationships with logging roads, both positive and negative, among Bornean felids [23,24] and we hypothesise that ridgelines may be preferentially selected by some Bornean felids to facilitate movement. We were interested in the possible difference between male and female Sunda clouded leopards, and their interactions with other felids, and so we undertook separate analyses for both sexes. We defined camera stations as being placed on logging roads and ridgelines via observation during camera deployment and by visually inspecting 30 m resolution topographical maps [40].

### Felid/Candidate prey co-occurrence all-subsets modelling

We sought to analyse the broad pattern of co-occurrence of biodiversity with each felid and use that to identify potential prey species based on spatial co-occurrence. We developed an all-subsets multivariate logistic regression model of felid occurrence as a function of the co-occurrence of a range of mammalian and avian species with each Bornean felid. First, we calculated the number of detections per camera station for each candidate prey variable, and we retained only those species or species groups with samples sizes of ≥ 20. Next, we developed all-subsets logistic regression models with Dredge function in the MuMIn library in R, predicting each felid species as a function of all combinations of the number of detections of candidate prey species co-occurring at camera stations. We produced final model averaged parameter values for the regression models predicting each felid species as functions of co-occurrence of potential prey.

### Temporal activity of felids and candidate prey

We used our photographic detection dataset to characterise the temporal activity patterns of sympatric Bornean felids and candidate prey species and to quantify the extent of temporal overlap between them. We followed the statistical approach developed by Ridout & Linkie (2009) [51] and performed all analyses in R using program Overlap [52,53]. We computed each species’ or species group’s terrestrial activity pattern independently using non-parametric von Mises kernel density estimation, which corresponds to a circular distribution [51], using the default smoothing value of 1.0. For the felid species, we also calculated the proportion of the density that lies within the dawn and dusk (denoted crepuscular), day (denoted diurnal), and night (denoted nocturnal) time periods. We defined dawn (05:00–07:00) and dusk (17:00–19:00) time periods as one-hour pre and post sunrise/sunset, and the intervening periods as day (07:00–17:00) and night (19:00–05:00). Lastly, we calculated a measure of overlap between Bornean felids and between the felids and all other candidate prey species using the coefficients of overlapping, Δ_1_ and Δ_4_, which range from 0 (no overlap) to 1 (complete overlap). Following the recommendation of Ridout & Linkie (2009) [51], we used their estimators Δ_1_ and Δ_4_ when the smaller of the two samples had <75 and ≥75 records, respectively. We obtained confidence intervals as percentile intervals from 10,000 smoothed bootstrap samples. We assessed the significance of temporal activity overlap by computing the 5th and 95th percentiles of the coefficients of overlapping (Δ_1_ and Δ_4_) values between all possible paired focal species.

We identified which species exhibited significantly low overlaps with Bornean felids and those which showed significantly high overlap patterns, which we defined as below the 5th percentile or above the 95th percentile of overlap across all candidate prey species, respectively. We also identified species which had relatively high and low overlaps with Bornean felids, which we defined as below the 10^th^ percentile and above the 90^th^ percentile of overlap across all species, respectively.

## Results

### Bornean felid detection dataset

The camera surveys yielded 2883 independent detections of Bornean felids (Table 2). Felid guild composition ranged from 4–5 species for five of the forest study areas, dropping to three at the higher elevation Crocker Range, and one within the highly fragmented and comparatively small Kabili-Sepilok. Sunda clouded leopards and marbled cats were recorded at all but one forest site, Kabili-Sepilok. Leopard cats were recorded at all forest sites and were the only felids to be recorded within the two oil palm plantation areas. In contrast, flat-headed cats were recorded within only two study areas, at two camera stations and on only four occasions, and thus were removed from subsequent analyses. Our survey efforts yielded 65,536 detections of non-felid animals, representing at least 120 species (57 mammals, 56 birds, and seven reptiles). Of these, 58 species or species groupings (35 mammals, six birds) had detection sample sizes ≥20 and were subsequently used in the candidate prey all-subsets and temporal activity modelling (S4 Table).

**Table 2:** Bornean felid detection data derived from intensive camera trap surveys of 10 study areas in Sabah, Malaysian Borneo.

Study area	No. independent captures ^a^ (Detection frequency ^b^)						
	Sunda clouded Leopard			Leopard cat	Bay cat	Marbled cat	Flat-headed cat
	pooled	males	females				
Danum Valley (1 & 2)	100 (1.032)	90 (0.928)	10 (0.103)	39 (0.402)	11 (0.113)	42 (0.433)	1 (0.010)
Tawau (1 & 2)	339 (0.969)	302 (0.863)	37 (0.106)	128 (0.366)	42 (0.120)	88 (0.251)	0
Crocker Range	51 (1.256)	46 (1.133)	5 (0.123)	28 (0.690)	0	11 (0.271)	0
Tabin	41 (0.634)	36 (0.557)	5 (0.077)	191 (2.956)	3 (0.046)	42 (0.650)	0
Ulu Segama	83 (2.915)	71 (2.494)	12 (0.421)	494 (17.352)	2 (0.070)	7 (0.246)	0
Malua	11 (0.284)	8 (0.207)	3 (0.078)	272 (7.032)	3 (0.078)	5 (0.129)	0
Kinabatangan	15 (0.730)	8 (0.389)	7 (0.341)	21 (1.022)	0	5 (0.243)	3 (0.146)
Kabili Sepilok	0	0	0	12 (0.276)	0	0 (0)	0
Danum Palm	0	0	0	624 (28.210)	0	5 (0.226) ^c^	0
Minat Teguh	0	0	0	164 (8.367)	0	0 (0)	0
Total	640	561	79	1973	61	205	4

^a^ Number of photographic captures of the same species or different individuals (patterned felids) per camera station, obtained ≥1 hour apart

^b^ Number of independent photographic captures per 100 camera trap nights.

^c^ one individual marbled cat was recorded walking along the edge of the forest, immediately adjacent to the plantation edge.

**Fig 2:**
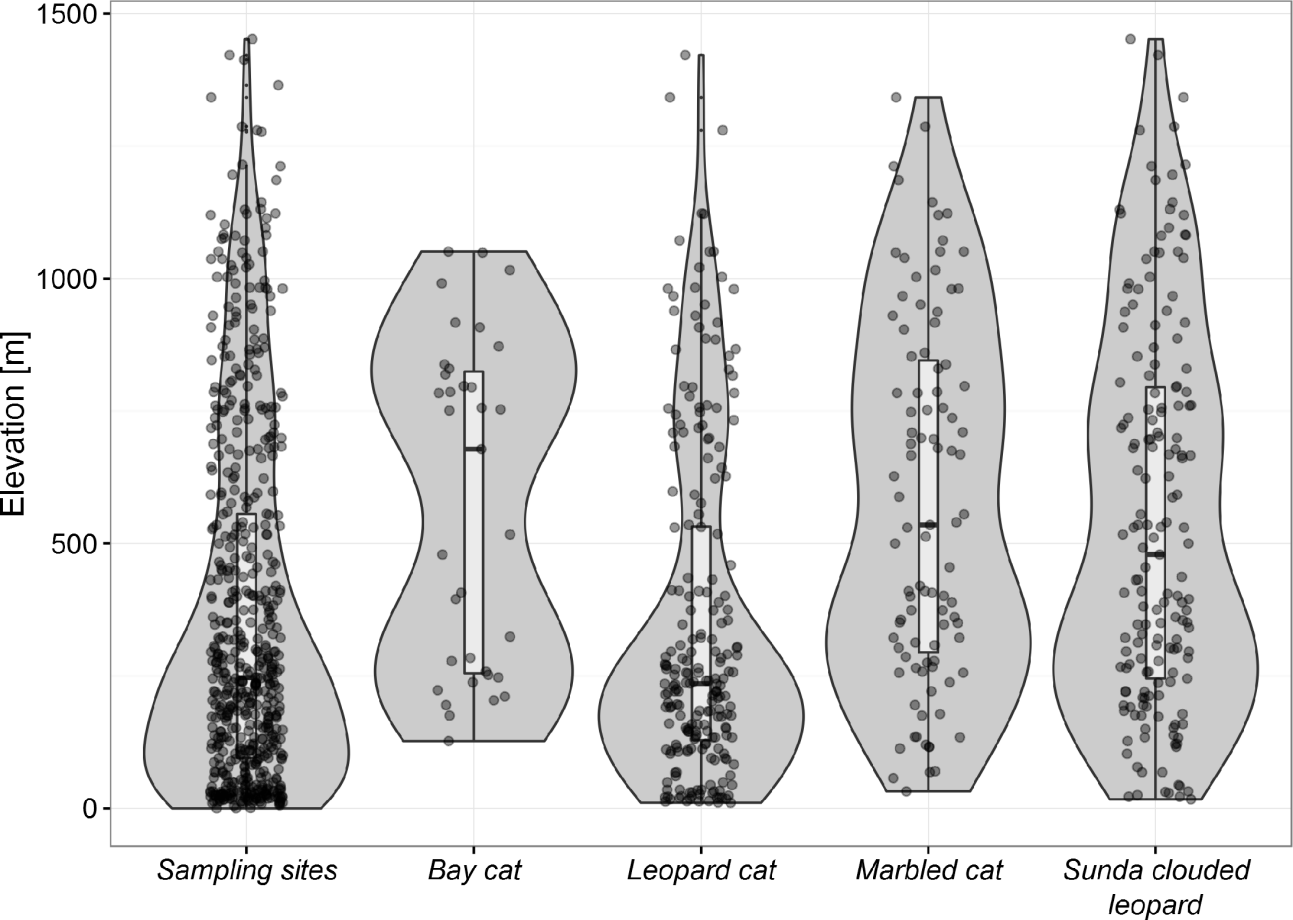
Violin plots displaying the elevational distribution over which Bornean felids were detected by camera traps. The diameter of the plot indicates the kernel density of the elevational range of each species. The boxplot shows the median elevational value and delineates the 25^th^ and 75^th^ percentile range for each species. The circular dots represent actual records of camera stations at which felids were detected and are arbitrarily assigned to either side of the midline.

Bornean felids varied in their use of the elevation range (Fig 2). Leopard cats were recorded from 10 to 1422 m, and comparison of leopard cat kernel density with that of the available camera stations suggests that they had a slight preference for lower elevation sites. Sunda clouded leopards and marbled cats shared a very similar elevational distribution and were recorded over much of the elevation range sampled, with Sunda clouded leopard recorded from 17 to 1452 m, and marbled cats from 32 to 1342 m. Kernel density estimates for both suggested a preference for higher elevation sites (600–1000 m). Bay cats were recorded over a more restricted elevational range, from 127 to 1051 m, and showed a bimodal kernel density distribution, with peaks at around 250 m and 800 m. We recorded flat-headed cats only in the lowlands, at 18 and 180 m.

### Multi-scale habitat modelling

The univariate scaling analyses showed that the strength of the relationship between Bornean felid occurrence and predictor habitat variables was highly dependent on the spatial scale at which each variable is derived, and that directionality of the relationship was reversed under some focal scales (S1 and S2 Figs; S5 Table). Following the univariate scaling and variable correlation analyses there were 13, 14, 14, and 13 final habitat covariates for Sunda clouded leopard, bay cat, marbled cat and leopard cat, respectively, for use in the multivariate analyses.

The final multivariate models showed that Bornean felids differed in their respective relationships with habitat covariates (Table 3). We refer to responses at the bottom third of our spatial scales analysed (ie 120 and 240 m) as fine scale, and those at the middle (480, 960, and 1920 m) and upper thirds (3840 and 7680 m) as moderate and broad scale, respectively. Sunda clouded leopards were most closely associated with areas with high levels of dipterocarp forest at relatively fine spatial scales, at high elevations within broad landscapes with low levels of human footprint, tree cover, and areas of plantation and scrub lands at broad spatial scales. Bay cats were most closely associated with areas of low fragmentation and low human footprint at relatively broad scales. Marbled cats were associated with areas of higher elevation dipterocarp and limestone forest at fine scales, with rough topography at the moderate scale and with low levels of fragmentation, human footprint, and areas of oil palm and scrub lands at relatively broad scales. The habitat variables influencing leopard cat occurrence differed starkly from the other three felids. Leopard cats were associated with areas of oil palm plantations at relatively fine spatial scales, at lower elevations at relatively broad scales, and with low levels of mosaic and regrowth areas at broad scales.

**Table 3:** Final multivariate models predicting the occurrence of four Bornean felids as a function of environmental predictor variables.

Species/variable	Optimal scale (m)	β	Adjusted SE β	z value	AIC imp.	p-value	
*Sunda clouded leopard*							
(Intercept)	-	-2.2348	0.2573	8.687		< 0.001	***
Effort	-	0.0077	0.0015	5.036	1	< 0.001	***
Human footprint	7680	-0.7342	0.2138	3.433	1	< 0.001	***
SFD: Lowland Mixed Dipt. forest	480	0.9610	0.2842	3.381	1	< 0.001	***
Elevation	120	0.4482	0.1395	3.212	1	0.0013	**
Tree cover	7680	-0.6267	0.2446	2.562	1	0.0104	*
Miet: Plantation/regrowth	7680	-0.3998	0.1672	2.391	1	0.0168	*
Gav: Agroforest/forest regrowth	7680	0.0802	0.1695	0.473	0.29	0.6359	
*Bay cat*							
(Intercept)	-	-5.3178	0.9891	5.376		< 0.001	***
Effort	-	0.0080	0.0020	4.012	1	< 0.001	***
Tree cover SD	960	-3.1656	1.9542	1.62	1	0.1050	
Human footprint	3840	-0.2944	0.2810	1.048	0.71	0.2950	
Roughness	7680	-0.0846	0.3474	0.244	0.49	0.8080	
*Marbled cat*							
(Intercept)	-	-2.8765	0.2912	9.877		< 0.001	***
Effort	-	0.0061	0.0016	3.863	1	< 0.001	***
SFD: Lowland mixed Dipt. forest & limestone veg.	120	0.4305	0.1251	3.441	1	< 0.001	***
Tree cover SD	3840	-0.5979	0.2805	2.132	0.93	0.0330	*
Roughness	960	0.4234	0.2033	2.083	0.85	0.0372	*
Human footprint	7680	-0.4000	0.2113	1.893	0.82	0.0584	
Miet: Plantation/regrowth	3840	-0.2668	0.2273	1.174	0.31	0.2404	
Elevation	120	0.1415	0.2073	0.682	0.19	0.4950	
Gav: Agroforest/forest regrowth	7680	-0.0772	0.2034	0.38	0.1	0.7042	
*Leopard cat*							
(Intercept)	-	-1.2990	0.2051	6.334		< 0.001	***
Gav: Oil palm plantations	480	0.9168	0.1563	5.866	1	< 0.001	***
Effort	-	0.0062	0.0014	4.469	1	< 0.001	***
Miet: Lowland mosaic	3840	-0.6901	0.1654	4.173	1	< 0.001	***
Elevation	1920	-0.3627	0.1401	2.589	1	0.0096	**
Miet: Plantation/regrowth	7680	-0.2487	0.1240	2.006	0.86	0.0448	*
Gav: Agroforest/forest regrowth	3840	0.1482	0.1350	1.098	0.34	0.2722	
SFD: Lowland mixed Dipt. forest & limestone veg.	7680	-0.1438	0.1748	0.823	0.26	0.4106	
Tree cover SD	240	0.0489	0.1633	0.299	0.15	0.7648	
Human footprint	960	-0.0142	0.1260	0.113	0.14	0.9103	

The optimal scale used for each variable is shown, alongside the Standardised regression coefficients (β) and AIC importance for habitat variables with significant univariate relationship to Bornean felid occurrence.

*P ≤ 0.05

**P ≤ 0.01

***P ≤ 0.001

**Fig 3:**
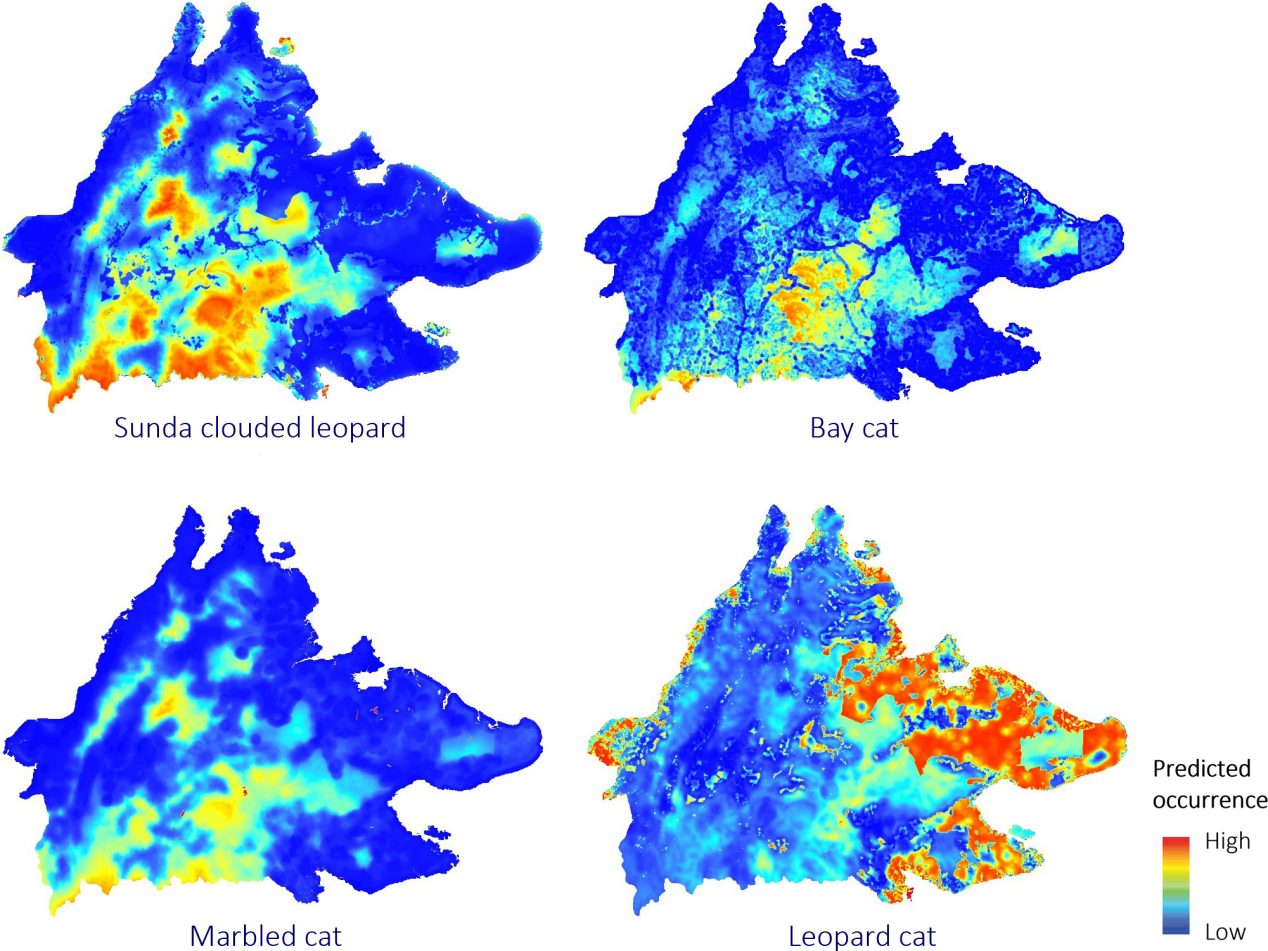
Maps showing the predicted occurrence of 4 species of Bornean felid based on multiscale habitat modelling.

The greatest differences in the spatial pattern of predicted occurrence were between the leopard cat and the other three felids, and the highest similarity in predicted occurrence was between marbled cat and clouded leopard, followed by marbled cat and bay cat (Fig 3). Pearson’s correlation between predicted probability of occurrence values and absolute differences in probability of occurrence across the surfaces strongly support this view (Table 4).

**Table 4:** Relationship between occurrence probability map predictions for four Bornean felids.

	Bay Cat	Leopard Cat	Marbled Cat	Clouded Leopard
Bay Cat	X	0.326	0.142	0.224
Leopard Cat	-0.214	x	0.304	0.356
Marbled Cat	0.7242	-0.2315	x	0.104
Clouded Leopard	0.6617	-0.3075	0.878	x

Lower triangle shows Pearson’s correlation between probability of occurrence values across the surfaces. Upper triangle is the mean absolute difference in probability of occurrence across the surfaces.

Sunda clouded leopards and marbled cats showed a broadly similar predicted distribution pattern (Fig 3). All of the relatively large and more contiguous forest patches were associated with higher predicted occurrence of these felids, and occurrence tended to be highest within the mid to higher elevation forest areas, and interior areas away from the forest patch edges. The areas of severely degraded logged forest and agroforest/forest regrowth along the western coastal region, and the oil palm plantation dominated areas in the east presented the lowest predicted occurrence for both Sunda clouded leopard and marbled cat. Sunda clouded leopards, however, exhibited low to moderate predicted occurrence in a number of relatively small forest patches, such as the Kinabatangan, whereas marbled cat occurrence was predicted to be very low in these areas, and the occurrence of clouded leopards was more positively influenced by higher elevation. As with Sunda clouded leopards and marbled cats, bay cats showed a very low predicted occurrence throughout all non-forest areas, but unlike the two other felids, areas of moderate to high levels of occurrence for the bay cat were primarily restricted to the core central forest area. In stark contrast to the three other felids, leopard cats were predicted as having very high occurrence throughout the oil palm dominated lowland landscape in eastern Sabah, and only moderate predictions of occurrence within the more heavily forested regions.

Bornean felids exhibited large differences in their respective use of roads and ridgelines (Table 5). Male Sunda clouded leopard occurrence was positively associated with roads and strongly and positively associated with ridgelines. Conversely, both female Sunda clouded leopard and bay cat occurrence was unrelated to either roads or ridgelines. Leopard cat occurrence was strongly and positively associated with roads but no relationship was found with ridgelines, whereas marbled cat occurrence was unrelated to roads but strongly and positively associated with ridgelines.

**Table 5:** Univariate logistic regression results for Bornean felids’ association with roads and ridgelines.

	Variable	Coefficient	AIC	*p*-value
Clouded leopard (males)	Road	0.5826	605.01	0.0368
	Ridgeline	2.5840	493.06	< 2E^-16^
Clouded leopard (females)	Road	0.3595	363.32	0.386
	Ridgeline	0.5515	361.04	0.077
Bay cat	Road	0.6518	238.81	0.218
	Ridgeline	0.5065	238.59	0.198
Marbled cat	Road	-0.0175	485.64	0.962
	Ridgeline	1.6415	445.03	9.53E^-11^
Leopard cat	Road	2.7040	680.48	2.67E^-14^
	Ridgeline	0.0305	767.9	0.890

### Bornean felid temporal activity patterns

**Fig 4:**
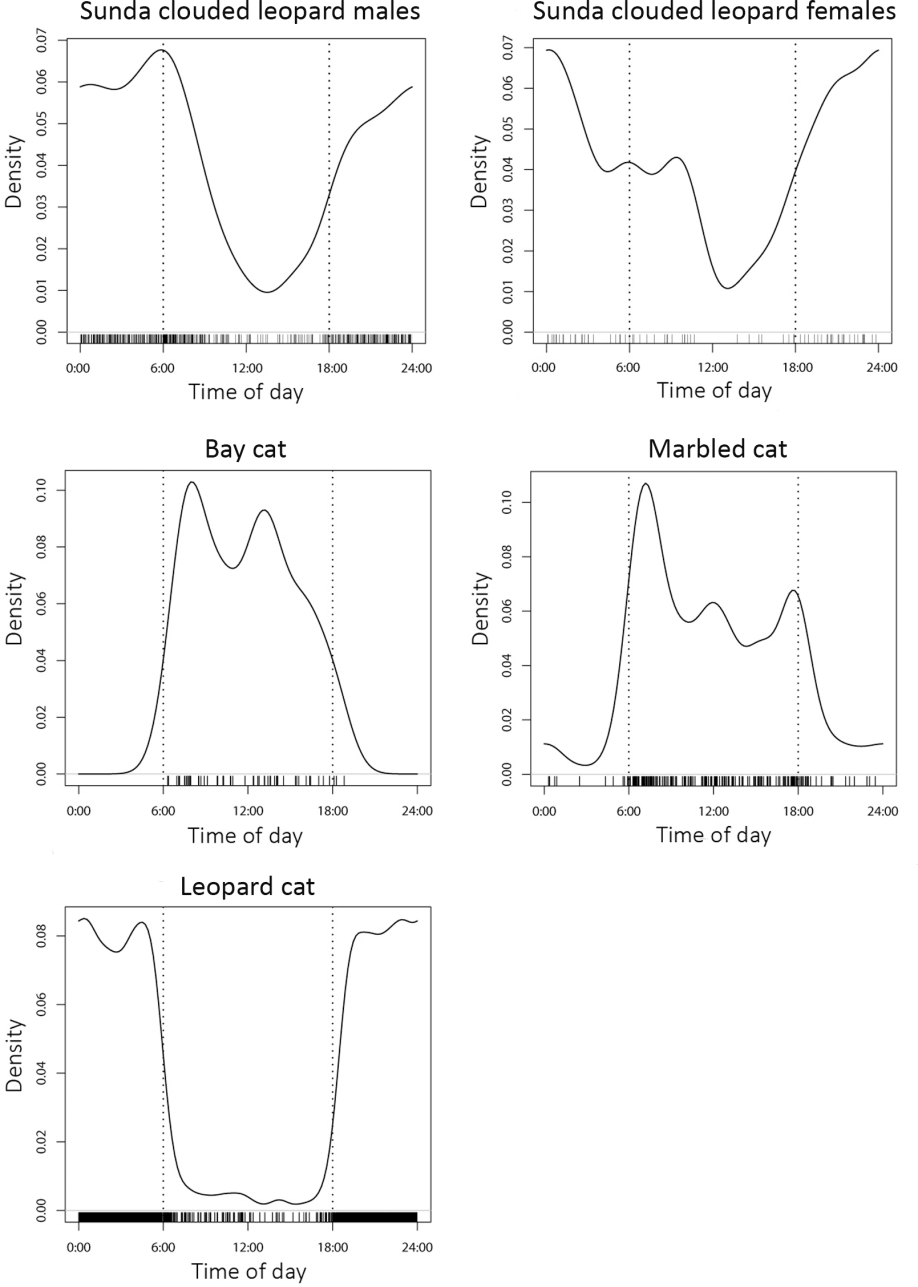
Temporal activity patterns of four sympatric Bornean felid species as estimated by kernel density estimates. a: Sunda clouded leopard males; b: Sunda clouded leopard females; c: bay cat; d: marbled cat; e: leopard cat. Activity data were derived from pooled camera trap surveys of ten study areas in Sabah, Malaysian Borneo. Dotted vertical lines indicate approximate times for dawn and dusk, and individual photographic detection times are indicated by the short vertical lines above the x-axis.

Bornean felids varied greatly in how they utilised the diel period (Fig 4; Table 6). Male and female Sunda clouded leopards were the most cathemeral, but were particularly active at night and least active at midday. Males showed a clear peak in activity at dawn, whereas females did not, but instead exhibited a peak in activity around midnight. Bay cats and marbled cats exhibited strongly diurnal activity patterns, with both showing little nocturnal activity, particularly bay cats. Both species showed peaks in activity at dawn, and also around or just after midday, although the latter was more markedly so in the bay cat. Activity increased around the dusk period in marbled cats, and decreased sharply after sunset, whereas activity in bay cats fell steadily after peaking during the middle of the day. Leopard cats were the most nocturnal of all the Bornean felids, and exhibited a sharp fall in activity before and during the dawn period, and a similarly sharp rise in activity during the dusk period. Low numbers of flat-headed cat photographic detections prohibited detailed analysis of activity, but of four records, two were at dawn, and two at night.

**Table 6:** Probability density mass of temporal activity of Bornean wild cats within four different time periods.

Species	Probability density mass for time period ^a^:			
	Dawn	Day	Dusk	Night
Sunda clouded leopard (males)	13.3 (6.6)	23.9 (2.4)	6.6 (3.3)	56.1 (5.6)
Sunda clouded leopard (females)	8.2 (4.1)	26.3 (2.6)	7.9 (4.0)	57.9 (5.8)
Leopard cat	9.3 (4.7)	4.0 (0.4)	6.2 (3.1)	81.0 (8.1)
Bay cat	8.5 (4.2)	81.0 (8.1)	7.8 (3.9)	2.3 (0.2)
Marbled cat	13.8 (6.9)	62.2 (6.2)	11.9 (6.0)	11.2 (1.1)

Probability density mass of temporal activity expressed as a percentage of the total mass; values within parentheses are the percentage of the total mass that each hour within the respective time-period contains.

^a^ Time periods are as follows: Dawn: 05:00–0700; Day: 07:00–17:00; Dusk: 17:00–19:00; Night: 19:00–05:00.

**Fig 5:**
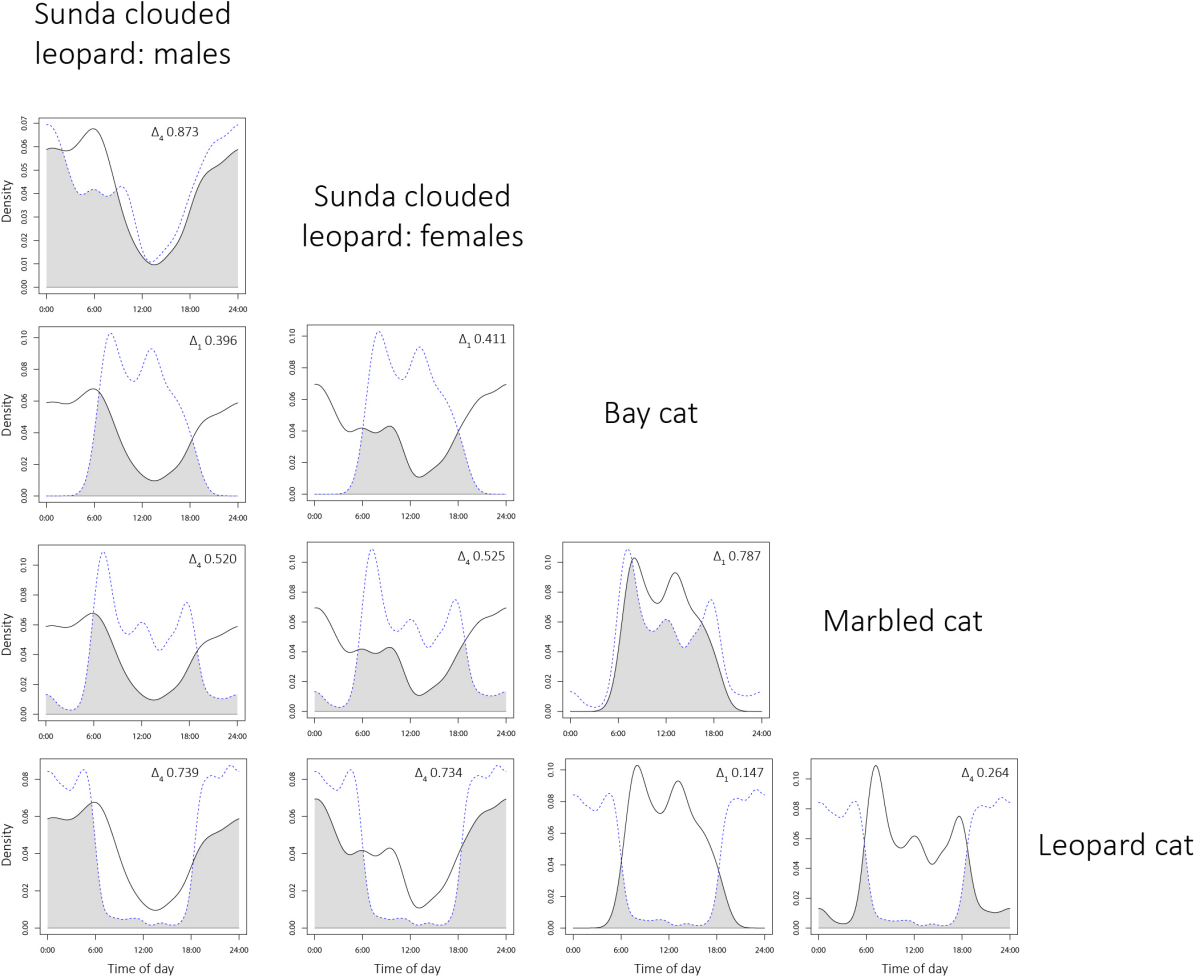
Overlaps of temporal activity patterns between Bornean wild cat species pairs, as estimated by kernel density estimates. The species in each column and each row are represented by solid lines and blue dotted lines, respectively. The coefficient of overlap (Δ_1_ and Δ_4_) is shown by the grey shaded areas which represent the overlap periods.

High levels of temporal overlap were exhibited between male and female Sunda clouded leopards (Δ_4_ 0.873), and between leopard cats and both male (Δ_4_ 0.739) and female (Δ_4_ 0.734) Sunda clouded leopards (Fig 5). Temporal overlap between bay cats and marbled cats was also high (Δ_1_ 0.787), whereas leopard cats exhibited low overlap with both bay cats (Δ_1_ 0.147) and marbled cats (Δ_4_ 0.264) (Fig 5). Moderate levels of overlap were exhibited between bay cat and Sunda clouded leopard males (Δ_1_ 0.396) and females (Δ_1_ 0.411) and between marbled cat and Sunda clouded leopard males (Δ_4_ 0.520) and females (Δ_4_ 0.525) (Fig 5).

### Temporal activity relationships between Bornean felids and potential prey

We calculated temporal overlaps between each Bornean felid and all candidate prey species or species groups (S6 Table). Male Sunda clouded leopards showed significant temporal activity associations (above 95^th^ percentile of overlap coefficient values) with sambar deer (*Rusa unicolor*), female clouded leopard and greater mousedeer (*Tragulus napu*), and relatively high temporal associations (above the 90^th^ percentile) with all mousedeer, masked palm civet (*Paguma larvata*) and leopard cat (S3 Fig). Male Sunda clouded leopards also showed significant temporal separation with pig-tailed macaque (*Macaca nemestrina*), tufted ground squirrel (*Rheithrosciurus macrotis*) and greater coucal (*Centropus sinensis*) (below the 5^th^ percentile) and relatively high temporal separation (below the 10^th^ percentile) with great Argus pheasant (*Argusianus argus*), long-tailed macaque (*M*. *fascicularis*) and emerald dove (C*halcophaps indica*) (S3 Fig).

Female Sunda clouded leopards showed similar temporal overlap relationships with candidate prey species as males, except that females also showed relatively high temporal associations with Malay civet (*Viverra tangalunga*), Hose’s civet (*Diplogale hosei*) and sun bear (*Helarctos malayanus*), and in females, pig-tailed macaques were below the 10^th^ percentile as opposed to below the 5^th^ (S3 Fig).

Bay cats showed significant temporal activity associations with short-tailed mongoose (*Herpestes brachyurus*), pig-tailed macaque, all small birds and bearded pig (*Sus barbatus*) juveniles, relatively high temporal associations with orangutan (*Pongo pygmaeus*) and blue-headed pitta (*Pitta baudii*), significant temporal separation with long-tailed (*Trichys fasciculata*) and common porcupines (*Hystrix brachyura*), and relatively high temporal separation with banded palm civet (*Hemigalus derbyanus*), rat spp., Malay badger (*Mydaus javanensis*) and common palm civet (*Paradoxurus hermaphroditus*) (S3 Fig). Furthermore, while not significant at the level we measured, temporal overlap coefficients were extremely high between bay cat and all partridges, all pheasants, Bulwer’s pheasant (*Lophura bulweri*), crested fireback (*Lophura ignita*), crested partridge (*Rollulus rouloul*) and great Argus pheasant (S6 Table).

Marbled cats showed significant temporal activity associations with all muntjac, Bornean yellow muntjac (*Muntiacus atherodes*), pig and pig adult, relatively high temporal associations with red muntjac (M*untiacus muntjak*) and crested fireback, significant temporal separation with long-tailed macaque and rat spp., and relatively high temporal separation with Malay badger, banded palm civet, pangolin (*Manis javanica*) and otter civet (*Cynogale bennettii*) (S6 Table; S3 Fig). Leopard cats showed significant temporal activity associations with Malay civet, common palm civet, Hose’s civet and moonrat (*Echinosorex gymnura*), relatively high temporal associations with thick-spined porcupine (*Hystrix crassispinis*) and banded linsang (*Prionodon linsang*), significant temporal separation with greater coucal, tufted ground squirrel and pig-tailed macaque, and relatively high temporal separation with great Argus pheasant, emerald dove and long-tailed macaque.

### Felid/candidate prey co-occurrence all-subsets modelling

The all-subsets logistic regression identified species that were associated with the occurrence of Bornean felids (Table 7). Four species showed a *p*-value of ≤0.05 and an AIC relative weight of 1 in relation to male Sunda clouded leopard occurrence, including Malay civet, common porcupine, sun bear and mongoose spp. All species had a positive coefficient, suggesting that they tend to occur at the same locations as male Sunda clouded leopards. Only one variable, sun bear, had a *p*-value of <0.05 in relation to female Sunda clouded leopard occurrence, again with a positive regression coefficient, indicating co-occurrence.

**Table 7:** Results of an all-subsets logistic regression analysis to predict the occurrence of Bornean felids as a function of potential prey species.

		AICc importance	Β	Adjusted SE β	z-score	p-value	
clouded leopard (males)	(Intercept)		-2.4610	0.2266	10.86	0.0000	***
	Malay civet	1.00	0.0408	0.0093	4.395	0.0000	***
	Common porcupine	1.00	0.0615	0.0187	3.291	0.0010	***
	Sunbear	1.00	0.1393	0.0442	3.15	0.0016	**
	Mongoose spp.	1.00	0.2178	0.1033	2.109	0.0349	*
	Banded linsang	0.89	0.2790	0.1464	1.906	0.0567	
	Pig	0.75	0.0085	0.0050	1.693	0.0904	
	Malay badger	0.29	0.0501	0.0428	1.17	0.2420	
	Binturong	0.30	0.3044	0.2664	1.143	0.2531	
	Yellow throated marten	0.31	0.0865	0.0895	0.966	0.3339	
	Collared mongoose	0.16	0.0947	0.1062	0.891	0.3727	
	Masked palm civet	0.11	0.0614	0.1021	0.601	0.5476	
	Short tailed mongoose	0.07	-0.0141	0.0378	0.373	0.7090	
	Banded palm civet	0.07	-0.0061	0.0169	0.362	0.7171	
	Hoses civet	0.06	0.0182	0.0565	0.322	0.7472	
	Effort	1.00	0.0005	0.0016	0.285	0.7757	
clouded leopard (females)	(Intercept)		-3.2000	0.2764	11.578	0.0000	***
	Effort	1.00	0.0047	0.0017	2.801	0.0051	**
	Sunbear	1.00	0.0776	0.0331	2.342	0.0192	*
	Hoses civet	0.82	0.0978	0.0531	1.84	0.0657	
	Mongoose spp.	0.59	0.1430	0.0866	1.652	0.0986	
	Short tailed mongoose	0.36	0.0407	0.0278	1.462	0.1436	
	Common porcupine	0.34	0.0235	0.0184	1.276	0.2018	
	Malaycivet	0.22	0.0078	0.0075	1.032	0.3019	
	Malay badger	0.22	-0.0812	0.0864	0.939	0.3475	
	Binturong	0.13	0.2662	0.3120	0.853	0.3936	
	Collared mongoose	0.08	0.0556	0.1060	0.525	0.5998	
	Banded linsang	0.07	-0.0968	0.2128	0.455	0.6493	
	Pig	0.07	-0.0017	0.0054	0.309	0.7575	
	Masked palm civet	0.08	0.0316	0.1185	0.266	0.7899	
	Yellow throated marten	0.06	-0.0088	0.0578	0.153	0.8785	
	Banded palm civet	0.06	0.0013	0.0170	0.076	0.9392	
bay cat	(Intercept)		-4.5857	0.4259	10.767	0.0000	***
	Sambar deer	1.00	0.0334	0.0133	2.513	0.0120	*
	All pheasants	0.60	0.0201	0.0095	2.112	0.0347	*
	All mousedeer	0.72	0.0130	0.0062	2.087	0.0369	*
	Malay civet	0.91	0.0189	0.0091	2.08	0.0376	*
	Great Argus pheasant	0.44	0.0224	0.0116	1.931	0.0535	
	Common porcupine	0.59	0.0356	0.0218	1.635	0.1021	
	Effort	1.00	0.0038	0.0024	1.623	0.1047	
	Short tailed mongoose	0.59	0.0529	0.0365	1.449	0.1473	
	Greater mousedeer	0.33	0.0275	0.0201	1.367	0.1715	
	Lesser mouse deer	0.27	0.0187	0.0156	1.202	0.2293	
	Pig subadult	0.16	-0.0420	0.0515	0.815	0.4150	
	Mongoose spp.	0.19	-0.1145	0.1433	0.799	0.4240	
	Crested fireback	0.10	0.0152	0.0213	0.715	0.4745	
	Bornean yellow muntjac	0.06	0.0020	0.0048	0.413	0.6797	
Marbled cat	(Intercept)		-2.8396	0.2525	11.244	0.0000	***
	Pig-tailed macaque	1.00	0.0241	0.0091	2.656	0.0079	**
	Banded linsang	0.98	0.3102	0.1391	2.23	0.0258	*
	Malay civet	0.98	0.0163	0.0077	2.113	0.0346	*
	Mongoose spp.	0.96	0.2007	0.0969	2.072	0.0383	*
	Sunbear	0.97	0.0647	0.0338	1.912	0.0559	
	Common porcupine	0.77	0.0326	0.0179	1.824	0.0681	
	Hoses civet	0.52	0.0875	0.0558	1.568	0.1169	
	Maskedpalmcivet	0.31	0.1395	0.1028	1.357	0.1748	
	Binturong	0.34	0.3587	0.2814	1.275	0.2025	
	Pig	0.33	0.0061	0.0048	1.265	0.2060	
	Tufted ground squirrel	0.25	0.0906	0.0770	1.177	0.2393	
	Bulwer’s pheasant	0.14	0.0281	0.0320	0.877	0.3803	
	Banded palm civet	0.07	0.0103	0.0164	0.629	0.5291	
	All pheasants	0.06	0.0035	0.0061	0.58	0.5618	
	Effort	1.00	0.0010	0.0018	0.554	0.5799	
	Rat spp.	0.06	0.0051	0.0102	0.497	0.6195	
	Yellow throated marten	0.06	0.0259	0.0525	0.494	0.6212	
	Short tailed mongoose	0.06	0.0177	0.0389	0.456	0.6483	
Leopard cat	(Intercept)		-1.4863	0.2038	7.292	0.0000	***
	Pig tailed macaque	1.00	0.0321	0.0095	3.396	0.0007	***
	Common palm civet	1.00	0.2154	0.0655	3.289	0.0010	**
	All small birds	1.00	0.3250	0.1171	2.775	0.0055	**
	Common porcupine	1.00	0.0382	0.0164	2.326	0.0200	*
	Collared mongoose	1.00	0.3832	0.1794	2.136	0.0327	*
	All pheasants	0.51	-0.0256	0.0128	2	0.0455	*
	Malay civet	0.93	0.0135	0.0068	1.992	0.0464	*
	Greater mousedeer	0.74	-0.0297	0.0150	1.982	0.0475	*
	Elephant	0.88	0.0907	0.0473	1.916	0.0554	
	Great Argus pheasant	0.60	-0.0343	0.0180	1.906	0.0566	
	Emerald dove	0.92	-0.2480	0.1332	1.863	0.0625	
	Greater coucal	0.96	1.0237	0.5598	1.829	0.0674	
	Otter civet	0.90	0.4989	0.2778	1.796	0.0725	
	All mouse deer	0.39	-0.0093	0.0061	1.534	0.1251	
	Effort	1.00	0.0014	0.0014	0.998	0.3182	
	Pig	0.13	-0.0038	0.0046	0.818	0.4133	
	Sambar deer	0.07	0.0056	0.0116	0.484	0.6284	

β: standardised regression coefficients; AIC importance: weighted average of models that include prey species variable, weighted by AIC model weight.

*P ≤ 0.05

**P ≤ 0.01

***P ≤ 0.001

For the bay cat, there were five species with a *p*-value of ≤0.05, including sambar deer, all pheasants, all mousedeer and Malay civet. Of these, sambar deer and Malay civet had an AIC relative weight of over 0.9, and all mousedeer and all pheasants had a AIC relative weight of 0.72 and 0.60, respectively. All four prey variables had a positive coefficient, suggesting that they tend to occur at the same locations as bay cat.

For the marbled cat, there were four species with a *p*-value of ≤0.05 and an AIC relative variable importance of over 0.9, including pig-tailed macaque, banded linsang, Malay civet, and mongoose spp. All of these variables had positive coefficients indicating that they tend to be present at sites where marbled cat was also present.

For the leopard cat, there were six species with a *p*-value of ≤0.05 and a relative weight of 1, including pig-tailed macaque, common palm civet, all small birds, common porcupine, collared mongoose and Malay civet. Four additional prey species had AIC weight of over 0.9, greater coucal, Malay civet, emerald dove and otter civet, but their *p*-values exceeded 0.05. Greater mousedeer and all pheasants had *p*-values of ≤0.05, but their AIC relative weights were 0.74 and 0.51, respectively. Six of eight of these variables showed positive regression coefficients, meaning that they typically occurred at the same locations as leopard cat, the exception being all pheasants and greater mousedeer, which were negatively associated with leopard cat occurrence.

## Discussion

This study accumulated the largest Bornean felid detection dataset to date and revealed evidence of niche separation among all felid assemblage members (Table 8). Potential mechanisms of resource partitioning between individual small felids and the larger Sunda clouded leopard appeared to be as numerous as among the smaller felid members. Consequently, our study provided little support for our a priori hypotheses that resource partitioning would be most pronounced between the four smallest species, which exhibit closely overlapping body sizes and thus may encounter the highest levels of competition (Jaksic and Marone, 2007). However, consistent with our a priori hypothesis, we found evidence that small Bornean felids exhibited spatio-temporal niche-separation with the larger and competitively dominant Sunda clouded leopard, which may pose a predation risk. Below, we explore these mechanisms in the context of these core hypotheses, focusing on the four species which our dataset permits, but also drawing on previous studies of flat-headed cats.

**Table 8:** Potential mechanisms of resource partitioning and coexistence among species pairs from an assemblage of five species of wild felids on Borneo.

Species pair		No. axes		Broad-scale habitat	Fine scale habitat selection			temporal activity	Prey species
			Elevation		vertical strata	logging road	Ridge		
Clouded leopard ♂	Clouded leopard ♀	4			*	***	***		*
Clouded leopard ♂	Bay cat	5	***			***	***	***	**
Clouded leopard ♀	Bay cat	4	***		**			***	**
Clouded leopard ♂	Marbled cat	4			*	***		***	**
Clouded leopard ♀	Marbled cat	3					***	***	**
Clouded leopard ♂	Leopard cat	4	***	***			***		***
Clouded leopard ♀	Leopard cat	5	***	***	**	***			***
Clouded leopard ♂	Flat-headed cat	6	***	***	*	*	*		***
Clouded leopard ♀	Flat-headed cat	3	***	***	*				
**Bay cat**	**Marbled cat**	3			***		***		***
**Bay cat**	**Leopard cat**	5	***	***		***		***	***
**Bay cat**	**Flat-headed cat**	4	***	***				***	***
**Marbled cat**	**Leopard cat**	7	***	***	***	***	***	***	***
**Marbled cat**	**Flat-headed cat**	6	*	***	***		***	***	***

*possible mechanism

**likely mechanism

*** highly likely mechanism.

Species pairs in bold highlight pairs with very similar body sizes (ratio <2) which are hypothesised to exhibit the greatest interspecific competition.

### Partitioning along the spatial habitat axis

All Bornean felids showed evidence of partitioning along the spatial niche dimension, although the degree of spatial differentiation varied greatly among felid guild members. Spatial partitioning was lowest between Sunda clouded leopards and marbled cats, followed by marbled cats and bay cats, although fine-scale partitioning was evident, whereas leopard cats showed evidence of strong spatial partitioning from that of the other three felids.

Sunda clouded leopards and marbled cats both selected higher elevation forest habitats at fine scales, and exhibited negative relationships with human dominated, highly disturbed habitat types at broad scales. Unlike Sunda clouded leopards, however, marbled cats were associated with limestone forest at fine scales, and rough topography at moderate scales. This may indicate marbled cats select topographical micro-sites that are avoided by clouded leopards as a mechanism to avoid inter-specific contact and risk of intra-guild predation. Marbled cats also showed a strong association with forested ridgelines, but not logging roads, while male clouded leopards, but not females, were associated with both logging roads and ridgelines, again suggesting marbled cat avoidance of male clouded leopards, which pose the greatest predation risk. These findings are in accordance with those of Wearn et al. (2013) [23], who showed that detection probabilities along logging roads and skid trails in a highly degraded forest in Sabah were significantly higher for clouded leopards, and higher for marbled cats along skid trails only, but their sample sizes permitted only preliminary conclusions. Overall, our analyses suggest that marbled cats exhibit broad-scale habitat overlap with clouded leopards, but select different fine scale habitats and avoid areas that clouded leopards most strongly select.

Female clouded leopards also seem to have substantial fine-scale habitat partitioning with males of their species. Specifically, female clouded leopards use logging roads, but our analysis showed that they do not strongly select them. Furthermore, unlike males, females were not associated with ridgelines. Their differential habitat selection may be a mechanism to reduce interactions with males, but conceivably also because they do not have the requirements to traverse over such large areas as their male counterparts, which may exhibit territorial defence and mate guarding behaviours, typical in male felids (e.g., [54]).

The bay cat exhibited similar broad scale habitat selection as Sunda clouded leopards and marbled cats, selecting areas of low fragmentation and low human footprint at relatively broad scales. Unlike these other felids, however, bay cats did not select higher elevation forest and used neither roads nor ridgelines, indicating substantial habitat separation from marbled cats and clouded leopards, particularly male clouded leopards. Unlike bay cats, both marbled cats and Sunda clouded leopards appear to be adapted to arboreal movement and foraging (e.g., [20]). While no quantitative data are available, such arboreal activity could provide further fine-scale spatial segregation between these felids.

Contrastingly, and in accordance with our a priori hypothesis, leopard cats showed a clear association with disturbed habitats, selecting oil palm plantations at relatively fine spatial scales and exhibiting a preference for lower elevations and areas with low levels of mosaic and regrowth at broad scales. Mohamed et al. (2013) [24] showed that leopard cat encounter rates from off-road camera traps were only 3.6–9.1% of those for on-road traps and their occupancy models revealed that canopy closure and ratio of climax to pioneer trees had a significantly negative impact on leopard cat occurrence.

Consistent with our hypothesis, and with the results emerging from several studies that have assessed multi-scale habitat selection optimization [25,26], we show that Bornean felids select habitats at multiple spatial scales, typically avoiding poor quality habitats at coarse scales often far exceeding that of the animal’s home range, while selecting particular habitat features at fine scales.

Our maps of felid distribution will facilitate the development of spatially explicit conservation recommendations and prioritization of areas for the threatened Bornean felids. Predicted occurrence of both Sunda clouded leopards and marbled cats reached a maximum within the higher elevation areas of the main contiguous forest block region in central Sabah, suggesting that this is a stronghold for these felids. The vast, heavily disturbed coastal areas and oil palm plantation dominated areas in the east presented the lowest predicted probability of occurrence for these felids. The maps highlight that two key protected areas in eastern Sabah which support Sunda clouded leopards, bay cats and marbled cats, namely the Tabin Wildlife Reserve and Tawau Hills Park, are predicted to be isolated from the populations in the core forest block. These predictions of distribution are broadly similar to those developed from presence only maximum entropy modelling [15–17], but our analyses are a methodological improvement on these earlier models as they provide information about responses to specific habitat variables and the relative influence of scale. Hearn et al. (2018) [37] used location data from GPS-tagged Sunda clouded leopards and showed that their movements were closely associated with forest with high canopy closure, and resisted by open habitats such as oil palm plantations. Our current study provides further evidence that highly degraded forest areas are possibly not used by Sunda clouded leopards.

The contrasting habitat preferences of leopard cats resulted in a predicted distribution that was almost completely non-overlapping those of the other three felids, with high probability of occurrence throughout the oil palm dominated lowland landscape in eastern Sabah, and only moderate predictions of occurrence within the more heavily forested regions. In contrast, Mohamed and Ross et al. (2016) [14] produced a presence-only predictive model of occurrence for this felid on Borneo that showed an island-wide, broadly even distribution across habitat types. The Mohamed and Ross et al. (2016) [14] model included an expert-opinion component to the assessment, which can be less reliable than empirically derived predictions of occurrence (e.g. [55]), in which forest habitats were classified as presenting similar habitat suitability to that of plantations. In contrast, our analysis strongly suggests that plantations present a much higher habitat suitability, a view supported by density estimates of this felid from Singapore (Chua et al., 2016). Our sampling of the oil palm landscape was restricted to areas bordering extensive forests (mean distance of camera station to forest: 4106 m, range 10–9100 m). It is possible, therefore, that leopard cat presence in plantations is linked to forest cover in the broader landscape, and that densities in the interiors of large plantations may be substantially lower. However, even plantations some distance from the major forest blocks in Sabah contain areas of natural (e.g., isolated forested steeply sloped hills) and semi natural (e.g., riparian strips of forest/scrub) habitats, which the leopard cats could utilise. Surveys from deeper inside large estates are needed to validate this prediction.

Perhaps the clearest evidence for spatial separation is shown by the flat-headed cat. As shown in this study, flat-headed cats are rarely recorded by camera traps, which likely reflects both the comparative rarity of these felids, but also their highly restricted habitat associations coupled with camera trap deployment strategies. Wilting et al. (2010, 2016) [10,11] showed that these felids were strongly associated with low-lying riverine and wetland habitats, indicating large divergence in habitat niche from the other fields in the Bornean guild.

Our maps of Bornean felid distribution include predictions for some regions that were beyond the sampled distribution. Nevertheless, our samples included a relatively large number of grids, in the key types of land use found in the state, and cover a wide range of ecological and topographical contexts. As such we believe the models are well resolved to make predictions across the extent of our analysis. It would be valuable, however, for future work to evaluate the accuracy of our predictions in areas that were not sampled in this study, such as the core of the large, forested mountain areas.

### Partitioning along the temporal axis

Both the bay cat and marbled cat exhibited highly overlapping diurnal patterns of activity, and thus showed little evidence of temporal partitioning. On the opposite end of the temporal spectrum, the activity patterns of the largely nocturnal male and female clouded leopards overlapped greatly both with one another, and with the highly nocturnal leopard cat, suggesting that they too did not differentiate along the temporal niche. Both bay cats and marbled cats, however, showed clear temporal partitioning with leopard cats and with clouded leopards. Our data regarding flat-headed cats did not permit statistical analysis, but the small number of camera trap detections and anecdotal sightings suggest this species is also nocturnal (e.g. Wilting et al., 2010), and thus temporal partitioning would further avoid competition with the diurnal bay cat and marbled cat, but not with the Sunda clouded leopard or leopard cat.

Thus, temporal partitioning may enable competitive displacement between clouded leopards and both marbled cats and bay cats, which have broadly similar spatial niches. Leopard cats are significantly smaller than clouded leopards, hence there is little dietary overlap, and their largely different habitat niches lead to little spatial overlap, and so no temporal displacement would be required to avoid competition among these species. The associations with highly disturbed and freshwater habitats exhibited by leopard cats and flat-headed cats, respectively, reduce the importance of temporal separation between them, but the presence of temporal divergence with the forest-associated bay cats and marbled cats contributes to further niche partitioning among these otherwise closely size-matched felids. However, the lack of temporal divergence between the similarly sized bay cats and marbled cats, which we have shown to also share large similarities in their spatial niche, means that perhaps their persistence is enabled by partitioning of prey.

### Differential use of prey

Co-occurrence is not evidence of a predator-prey relationship, and many co-occurring species will have no trophic association with one another. Our dataset and analyses, therefore, do not allow us to draw firm conclusions regarding Bornean felid prey choice. Nevertheless, for a predator-prey relationship to exist the two species must indeed co-occur. Our analysis demonstrates patterns of co-occurrence with a large number of species, which can be used to suggest possible predator-prey relationships which can be tested in future studies. Our results demonstrate that male Sunda clouded leopards co-occur with common porcupines, a species which they are thought to prey upon [56]. Significant spatial co-occurrence was also exhibited between male Sunda clouded leopard and Malay civet, sun bear and mongoose spp. which are unlikely candidate prey. This association may reflect mutual selection for similar habitat conditions, such as forest ridgelines, rather than any direct associative behaviour. Both male and female Sunda clouded leopards showed significant temporal overlaps with sambar deer and greater mousedeer. While adult sambar may exceed 200 kg, juveniles and subadults fall within the mass range that clouded leopard males are likely to take based on predator/prey mass allometries [54,57], but such theory predicts that only calves would be taken by female Sunda clouded leopards. Nevertheless, these data suggest that sambar may indeed constitute an important resource for these felids. Indeed, Mohamed et al. (2009) [58] reported that a juvenile sambar weighing an estimated 30–35 kg was killed and partially consumed by a Sunda clouded leopard. Interestingly, using a sub-set of the data examined in this paper, Ross et al. (2013) [28] showed that greater mousedeer exhibited shifts in temporal activity in a forest devoid of Sunda clouded leopards (Kabili-Sepilok), which, alongside our findings, suggests that they too may be important prey for Sunda clouded leopards. We found no evidence of co-occurrence or temporal synchronisation of Sunda clouded leopard males or females with that of bearded pigs, but previous studies have shown that they may indeed be important prey. Ross et al. (2013) [28] showed that bearded pigs exhibited a similar shift in activity in the absence of Sunda clouded leopards as that exhibited by greater mousedeer, and Mohamed et al. (2009) [58] detail an observation of a presumed male Sunda clouded leopard killing and subsequently dragging a 20–25 kg bearded pig up to the first storey of a wooden observation tower. These patterns of co-occurrence, in conjunction with previous studies showing temporal interaction among clouded leopards and these species, suggest there may be behavioural responses by clouded leopards to associate with these species in both space and time, potentially to maximize predation efficiency.

The bay cat remains one of the least known of the world’s wild cats, and to our knowledge there are no published data regarding these felids’ prey choices. We showed that bay cats exhibited significant spatial co-occurrence with all mousedeer and all pheasant’s species-groups, both of which are within the predicted size range of this felid based on predator/prey mass allometries [54,57]. Bay cats showed significant temporal activity associations with all small birds and blue-headed pitta, and, while not statistically significant, exhibited very high temporal overlap coefficients with a range of terrestrial, large and small bodied birds. Intriguingly, a bay cat was reportedly captured in Sarawak having been attracted to a captive pheasant enclosure [20]. The strength of these associations is highly suggestive of specialized predation focusing on ground-dwelling birds, which could provide a means for niche partitioning with marbled cats, allowing coexistence of these species that appear to be highly similar based on temporal and habitat selection niche axes. Further research on the diet composition of the bay cat is required to verify this hypothesis.

The marbled cat was shown to co-occur with pig-tailed macaque, banded linsang, Malay civet, and mongoose spp. Marbled cats have well documented arboreal adaptations (e.g., [20]), and so it is likely that these felids actively prey upon arboreal species, which could conceivably include pig-tailed macaques, perhaps targeting juveniles. In Thailand, a marbled cat was suspected of preying on a juvenile Phayre’s leaf monkey (*Trachypithecus phayrei*), which, at an estimated weight of around 5 kg, may have exceeded that of the cat [22]. The stomach contents of a female marbled cat shot on the ground at night in an old logged forest contained a small species of *Rattus* [21].

The diet of leopard cats is well studied and is thought to be comprised principally of murid rodents (e.g., [12,13,59], although, depending on geographical location, they will also take young ungulates, hares, birds, reptiles, insects, eels and fish [60]. We did not show evidence for associations with rodents in our study, but this may reflect the reduced ability of camera traps to detect small species.

Our analyses provide some of the first data regarding spatial and temporal associations with potential prey of Bornean felids, and provide tentative evidence that prey selection may vary among Bornean felids to enable co-existence. Similarity in spatial use and/or activity patterns between predator and prey is, by itself, not compelling evidence that a predator relies on any prey species, however. Indeed, felids such as jaguars and pumas may have activity patterns in phase with their main prey [6] or, as with lions, have cycles that oppose those of their prey [61]. Nevertheless, these analyses serve to highlight potential prey relationships, which can later be tested via analysis of the composition from stomach contents or scats.

## Supporting information

S1 Fig Scaling plots of AICc values and coefficient values for relationships between Bornean felid species’ occurrence and a range of predictor habitat variables cover type across 7 spatial scales. Spatial scales with the lowest AICc were deemed to be optimal, and used for further analysis.
(PDF)

S2 Fig Bar chart showing the optimal scaling of habitat variables in Bornean felids. (PDF)

S3 Fig Histogram of the frequency of coefficient of temporal overlap (Δ1 and Δ4) values between Bornean felids and all other species and species groups (n = 58). a. Sunda clouded leopard males; b. Sunda clouded leopard females; c. bay cat; d. marbled cat; e. leopard cat. The 5th and 95th percentiles of the Δ1 and Δ4 distribution for each felid are shown in vertical red lines, and the 10th and 90th percentiles are shown in vertical blue lines.
(PDF)

S1 Table Details of the eight forest and two oil palm plantation study areas in Sabah, Malaysian Borneo. (PDF)

S2 Table Details of camera trap survey protocols for surveys of eight forest areas and two palm oil plantations in Sabah, Malaysian Borneo. (PDF)

S3 Table Covariates used in the multi-scale habitat modelling of Bornean felid's occurrence in Sabah, including variable names, metrics calculated, and data sources. (PDF)

S4 Table Table of variables used in the felid/candidate prey co-occurrence all-subsets modelling and the felid/candidate prey temporal activity overlap analysis, showing variable description, number of independent photographic records for each species/group of species. (PDF)

S5 Table Results of univariate logistic regressions to assess the relative importance of habitat variables in predicting Bornean felid occurrence, showing optimal scale of each habitat variable. Variables showing p values <0.2 were used in the multivariate analyses.
(PDF)

S6 Table Table of overlaps of temporal activity patterns between Bornean wild cat species pairs and between Bornean wild cats and their potential prey species, as estimated by kernel density estimates. The coefficients of overlap (Δ1 and Δ4) are accompanied by the upper and lower values of the 95% confidence limits.
(PDF)
