# Supplementary material for: Spatio-temporal ecology of sympatric felids on Borneo. Evidence for resource partitioning?
Source: PLoS One. 2018 Jul 20;13(7):e0200828. doi: 10.1371/journal.pone.0200828 (PMC6054408; doi:10.1371/journal.pone.0200828)

Spatio-temporal ecology of sympatric felids on Borneo. Evidence for resource partitioning?

S1 Fig. Scaling plots of AICc values and coefficient values for relationships between Bornean felid species' occurrence and a range of predictor habitat variables cover type across 7 spatial scales. Spatial scales with the lowest AICc were deemed to be optimal, and used for further analysis.

(a) Sunda clouded leopard

Elevation

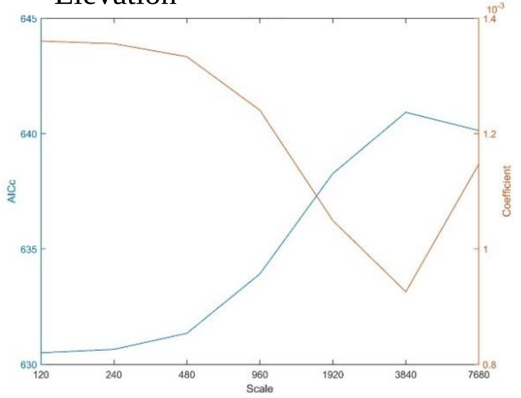

Gaveau: Agroforests / forest

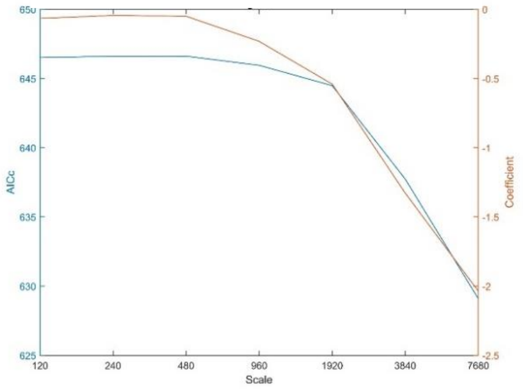

Gaveau: Oil palm plantations

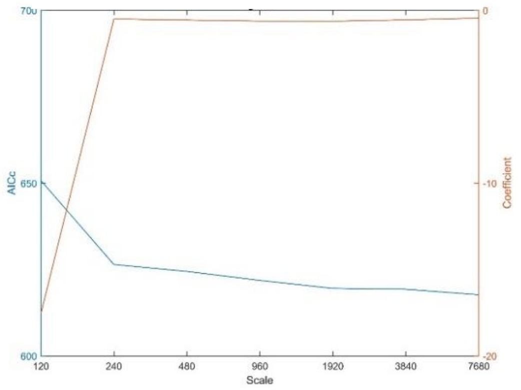

Gaveau: Logged forests

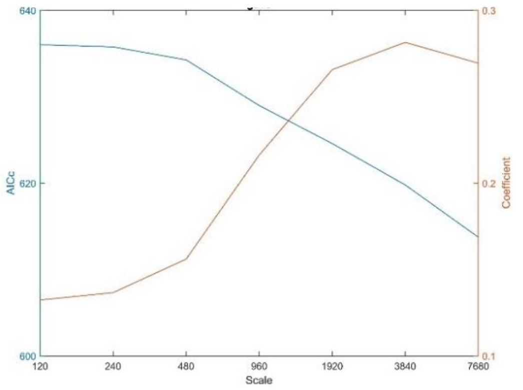

Miet 10

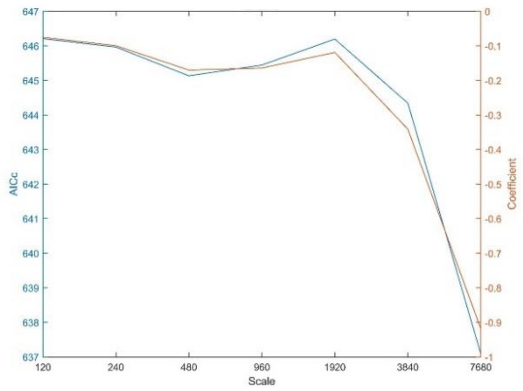

Miet 7

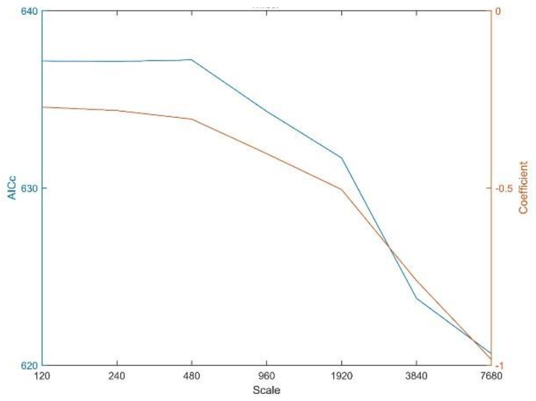

Miet 8

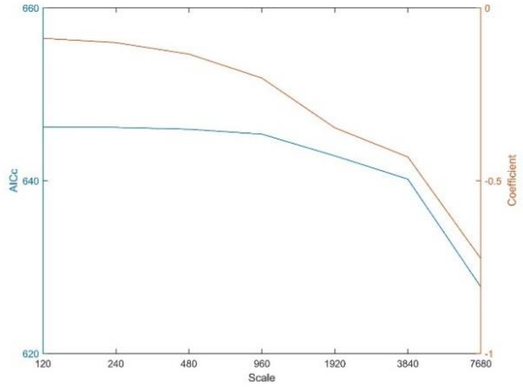

SFD 10

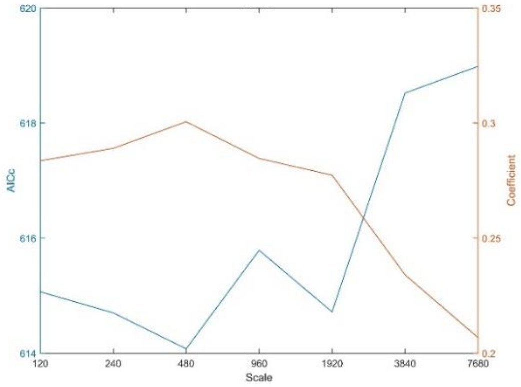

SFD 11

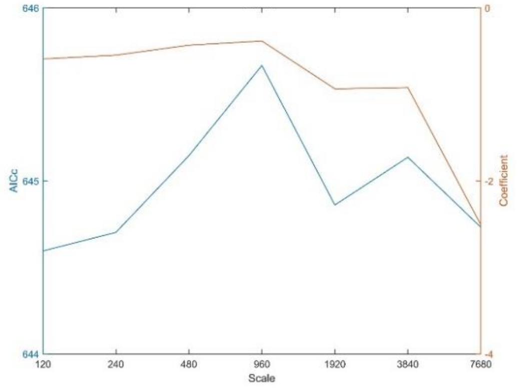

Human footprint

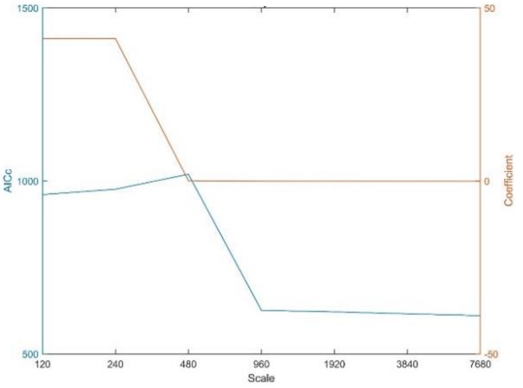

Topographical roughness

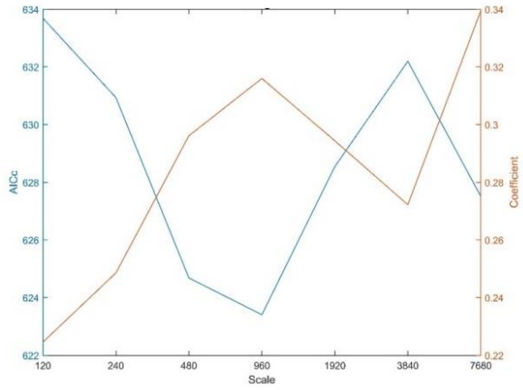

Tree cover

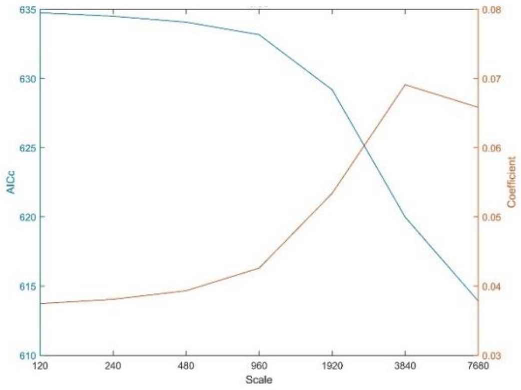

Tree cover SD

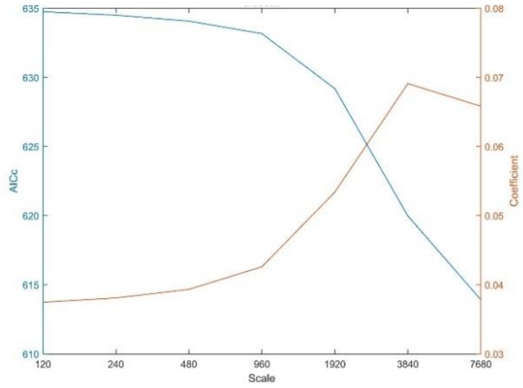

(b) Bay cat

Elevation

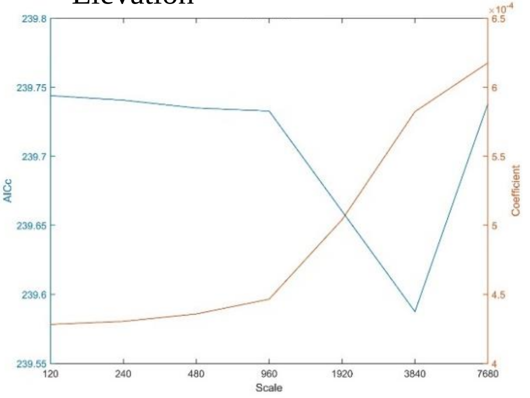

Gaveau: Agroforests / forest

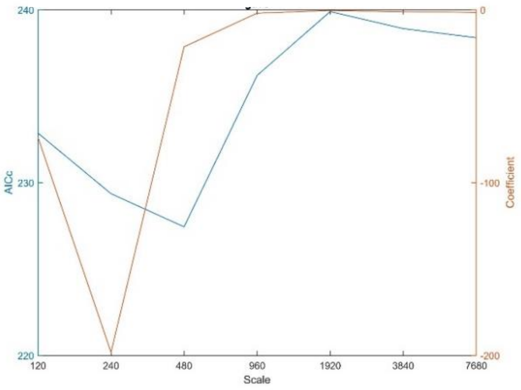

Gaveau: Oil palm plantations

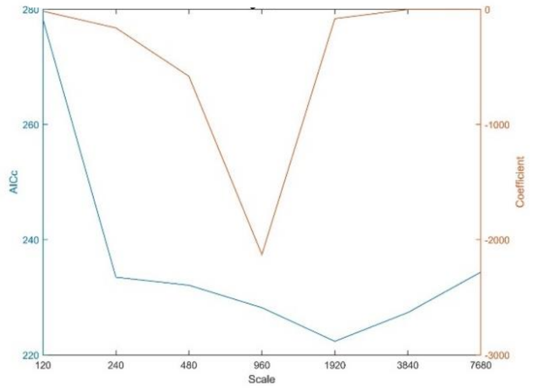

Gaveau: Logged forests

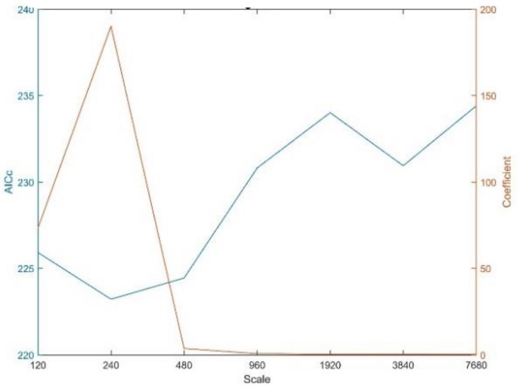

Meit 10

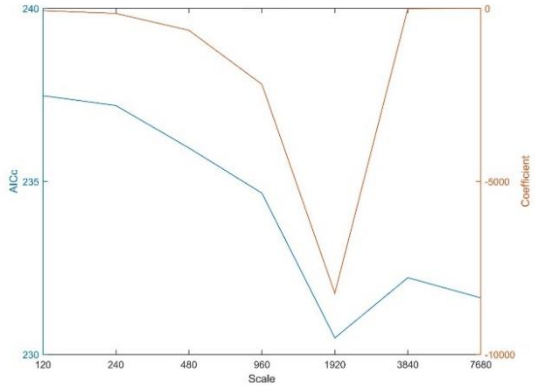

Meit 7

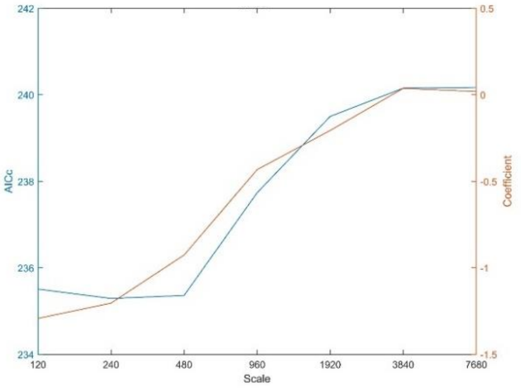

Miet 8

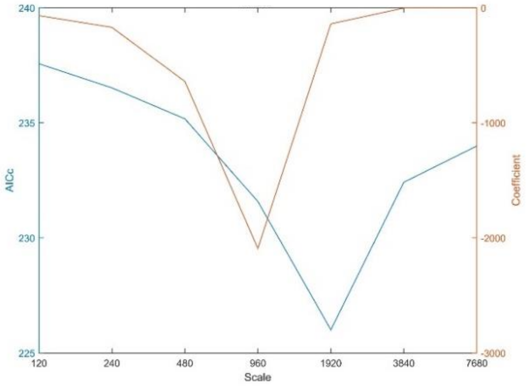

SFD 10

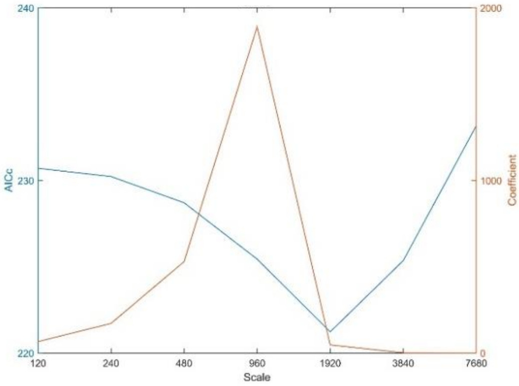

SFD 11

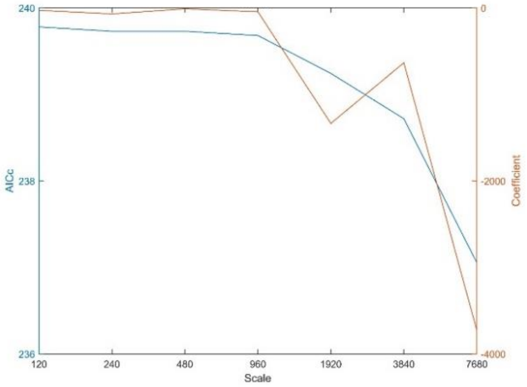

Human footprint

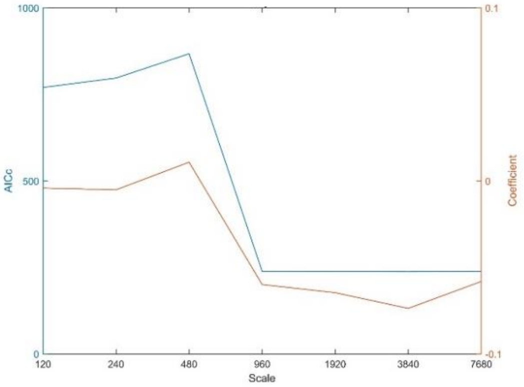

Topographical roughness

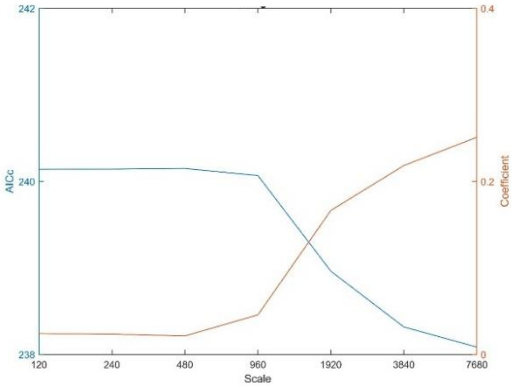

Tree cover

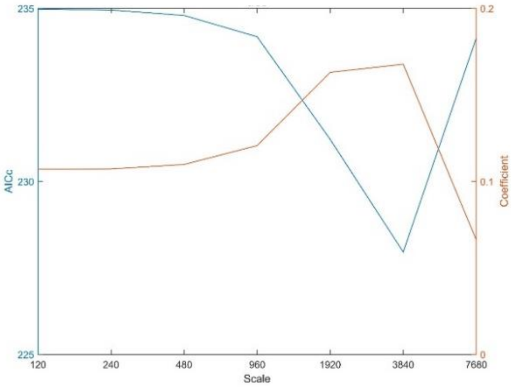

Tree cover SD

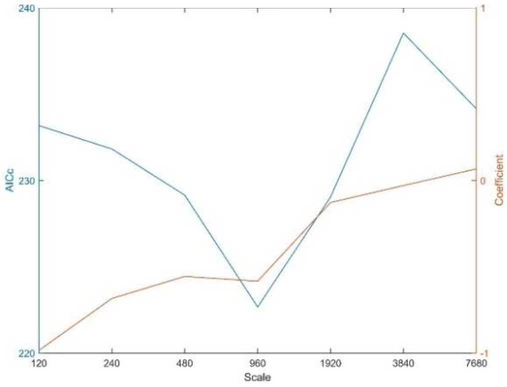

(c) Marbled cat

Elevation

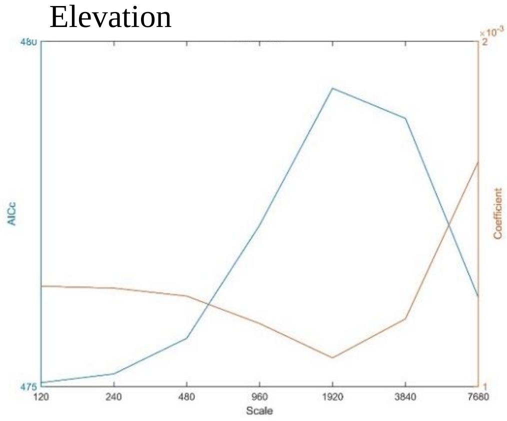

Gaveau: Agroforests / forest

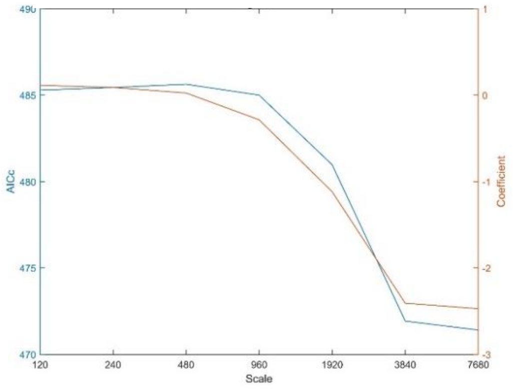

Gaveau: Oil palm plantations

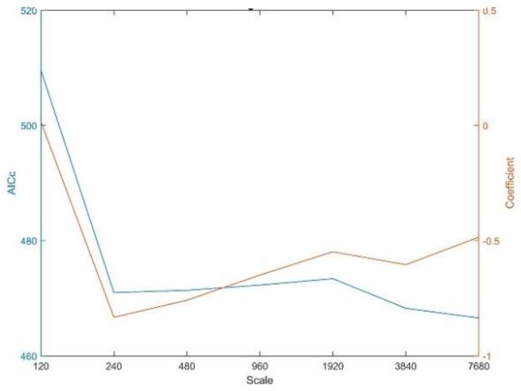

Gaveau: Logged forests

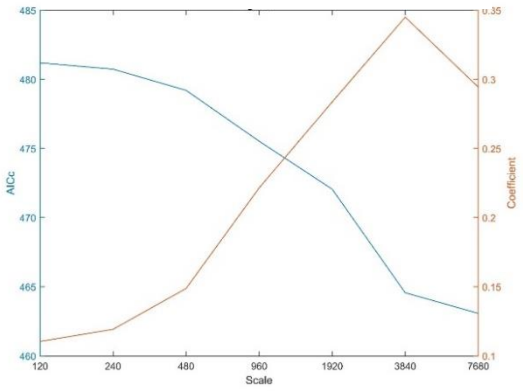

Miet 10

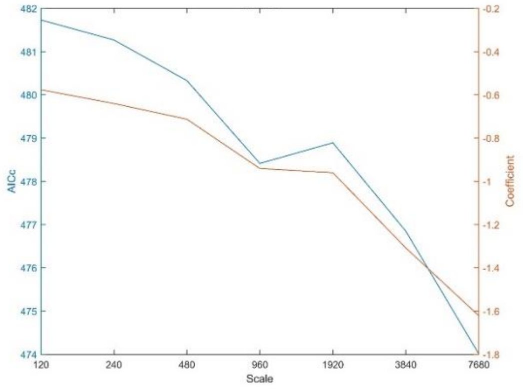

Miet 7

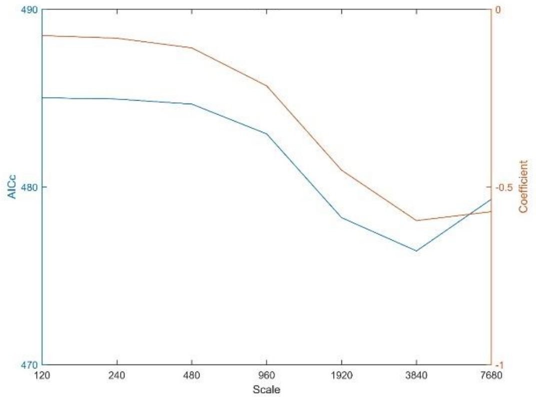

Miet 8

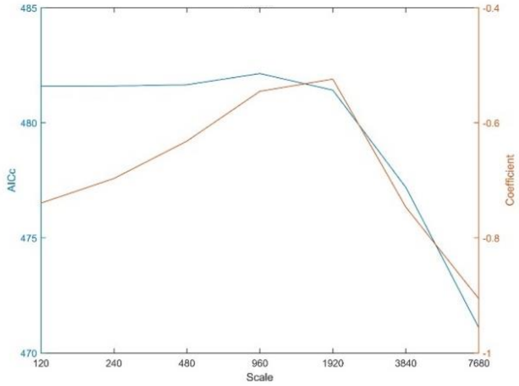

SFD 10

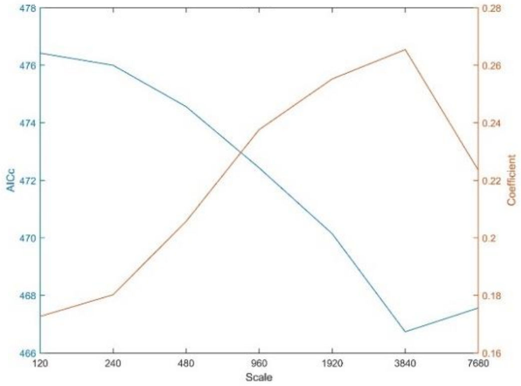

SFD 11

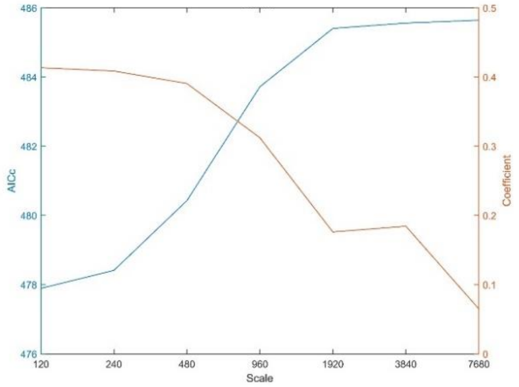

Human footprint

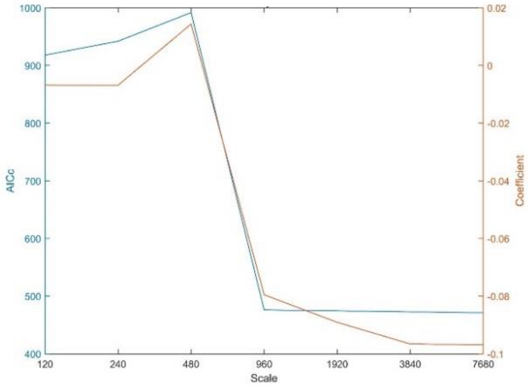

Topographical roughness

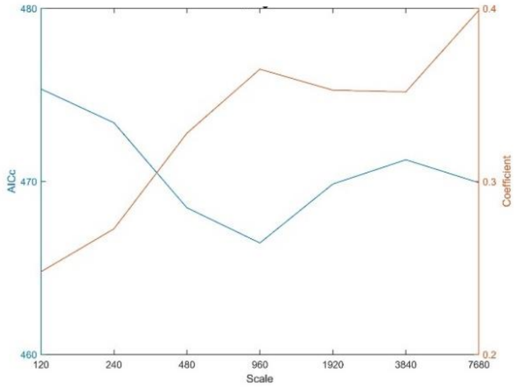

Tree cover

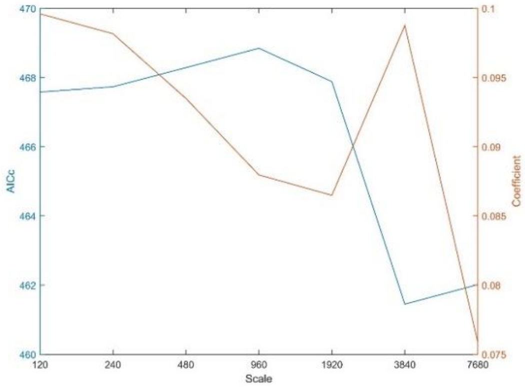

Tree cover SD

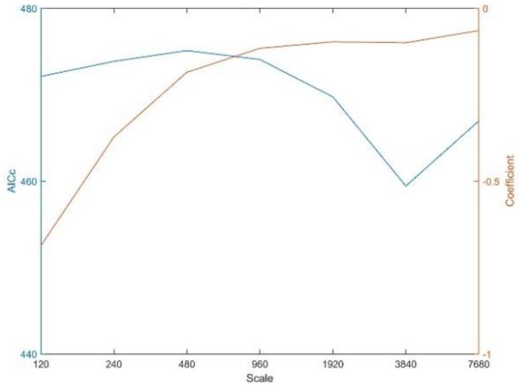

(d) Leopard cat

Elevation

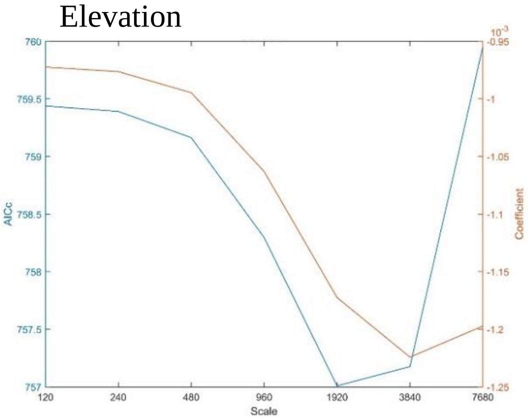

Gaveau: Agroforests / forest

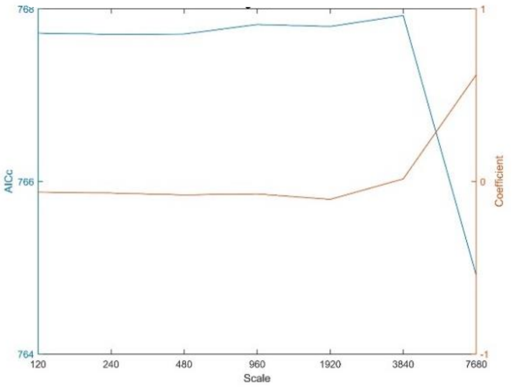

Gaveau: Oil palm plantations

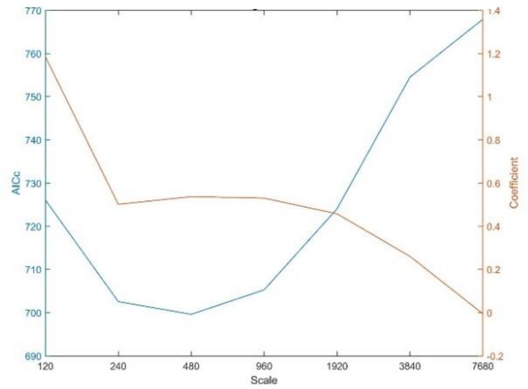

Gaveau: Logged forests

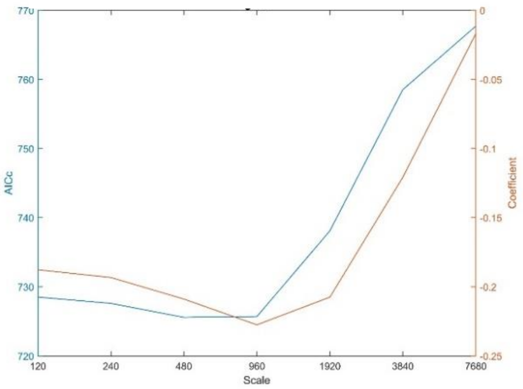

Miet 10

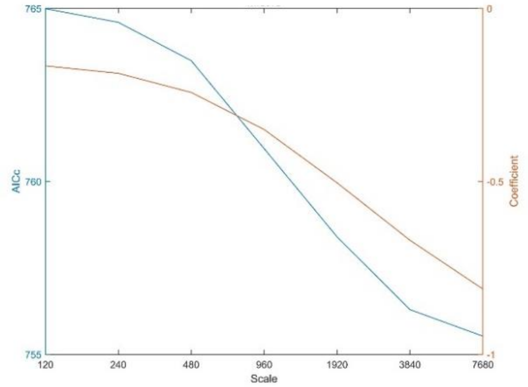

Miet 7

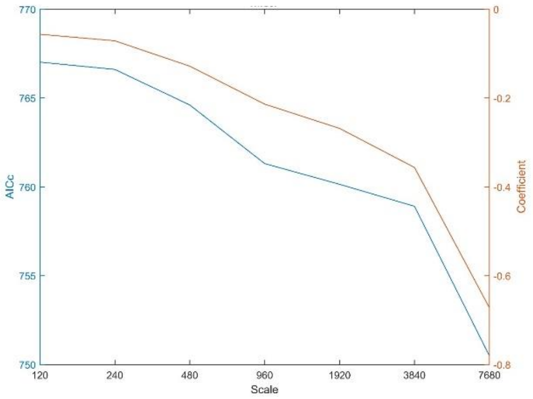

Miet 8

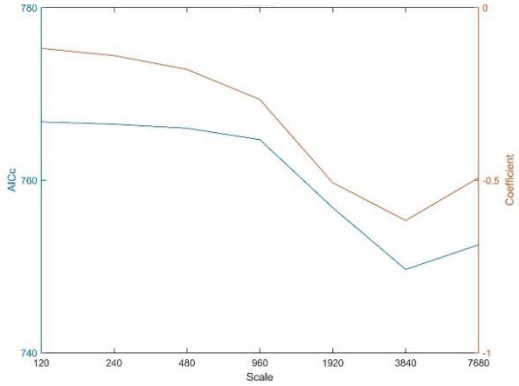

SFD 10

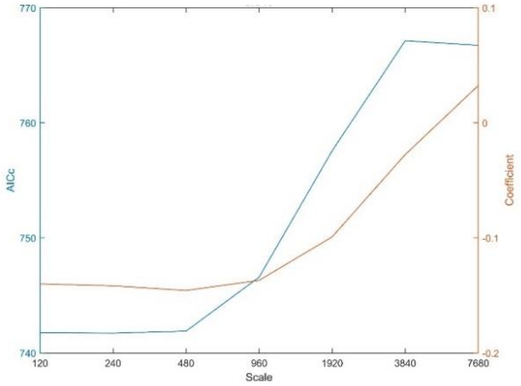

SFD 11

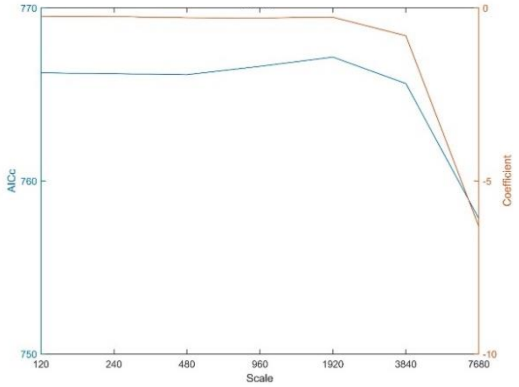

Human footprint

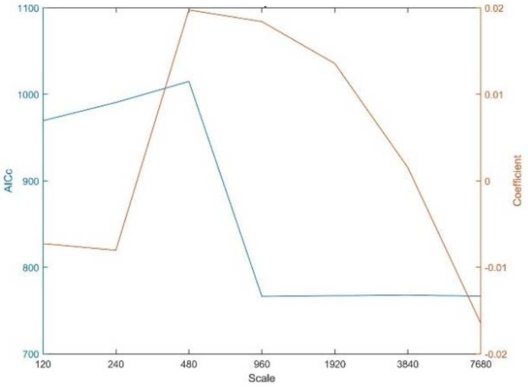

Topographical roughness

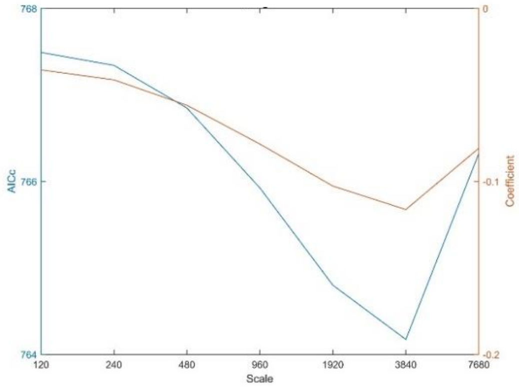

Tree cover

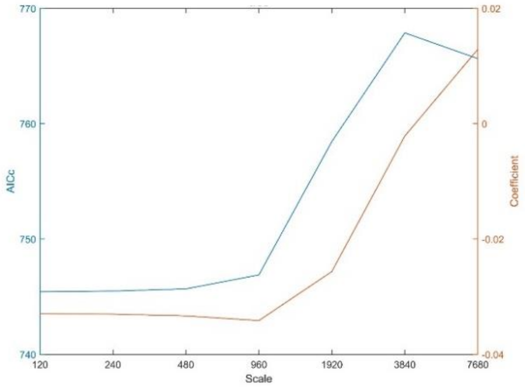

Tree cover SD

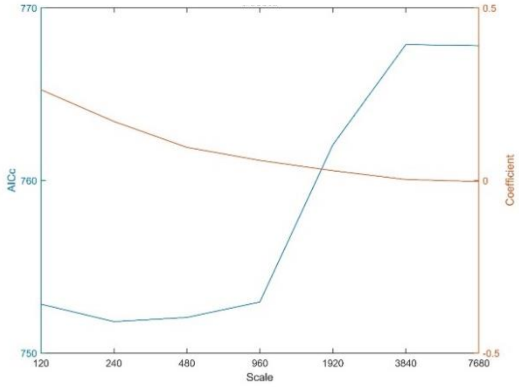

Supplement: S1 Fig — Spatial scales with the lowest AICc were deemed to be optimal, and used for further analysis. (PDF) [file pone.0200828.s001.pdf]
