# Supplementary material for: Spatio-temporal ecology of sympatric felids on Borneo. Evidence for resource partitioning?
Source: PLoS One. 2018 Jul 20;13(7):e0200828. doi: 10.1371/journal.pone.0200828 (PMC6054408; doi:10.1371/journal.pone.0200828)
Supplement: S2 Fig — (PDF) [file pone.0200828.s002.pdf]

Spatio-temporal ecology of sympatric felids on Borneo. Evidence for resource partitioning?

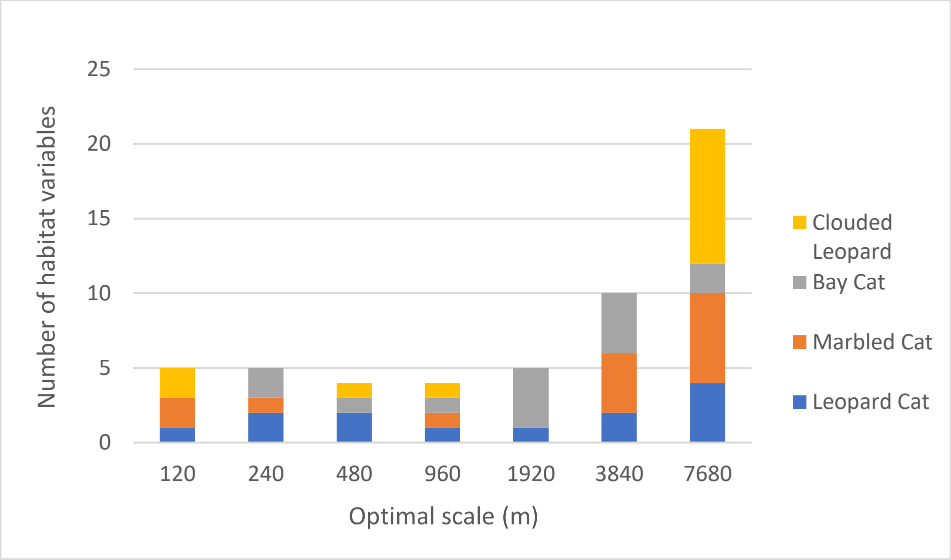

S2 Fig. Bar chart showing the optimal scaling of habitat variables in Bornean felids.
