# Supplementary material for: Spatio-temporal ecology of sympatric felids on Borneo. Evidence for resource partitioning?
Source: PLoS One. 2018 Jul 20;13(7):e0200828. doi: 10.1371/journal.pone.0200828 (PMC6054408; doi:10.1371/journal.pone.0200828)
Supplement: S3 Fig — a. Sunda clouded leopard males; b. Sunda clouded leopard females; c. bay cat; d. marbled cat; e. leopard cat. The 5th and 95th percentiles of the Δ1 and Δ4 distribution for each felid are shown in vertical red lines, and the 10th and 90th percentiles are shown in vertical blue lines. (PDF) [file pone.0200828.s003.pdf]

Spatio-temporal ecology of sympatric felids on Borneo. Evidence for resource partitioning?

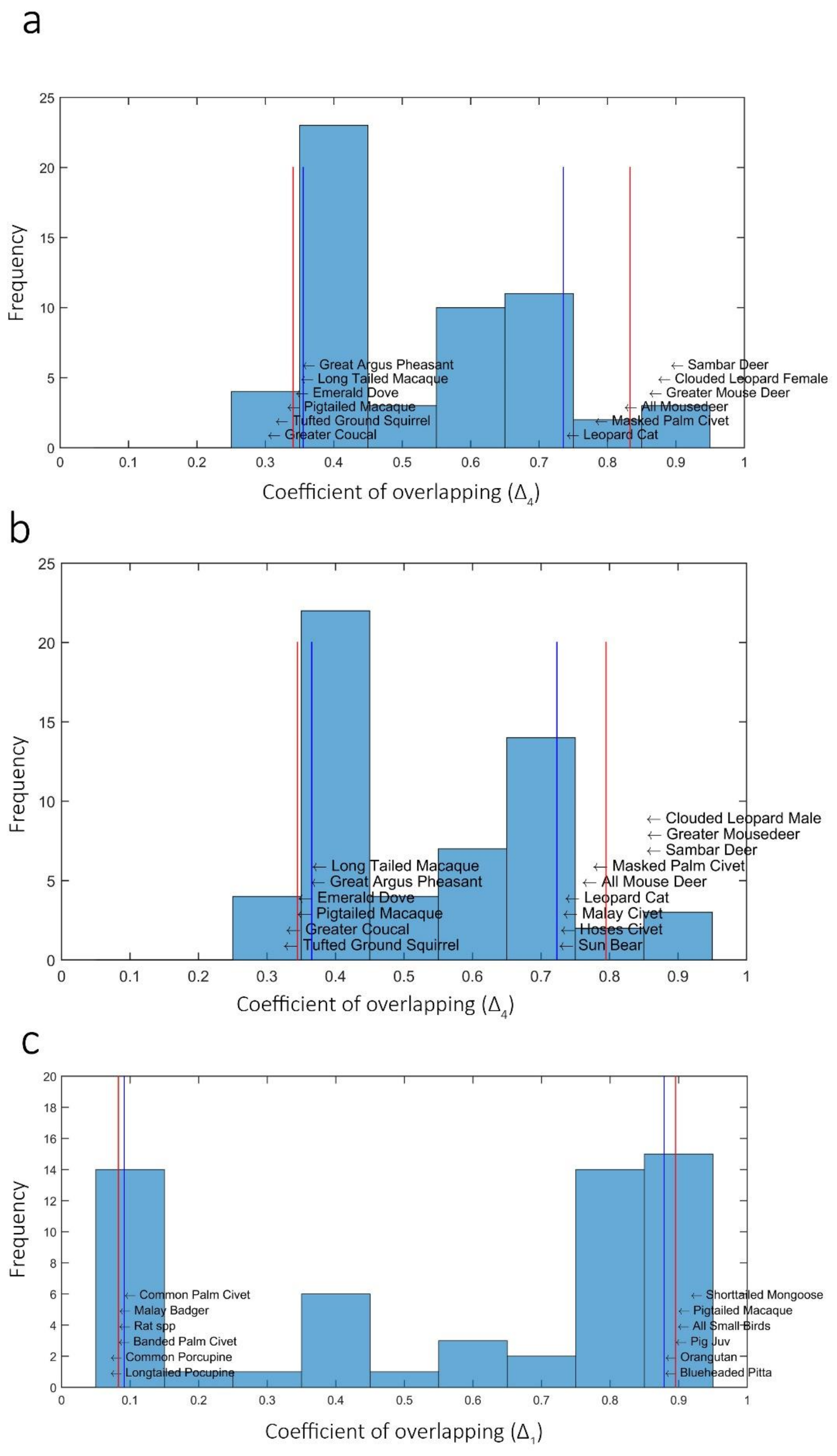

S3 Fig. Histogram of the frequency of coefficient of temporal overlap (Δ1 and Δ4) values between Bornean felids and all other species and species groups (n= 58). a. Sunda clouded leopard males; b. Sunda clouded leopard females; c. bay cat. The 5th and 95th percentiles of the Δ1 and Δ4 distribution for each felid are shown in vertical red lines, and the 10th and 90th percentiles are shown in vertical blue lines.

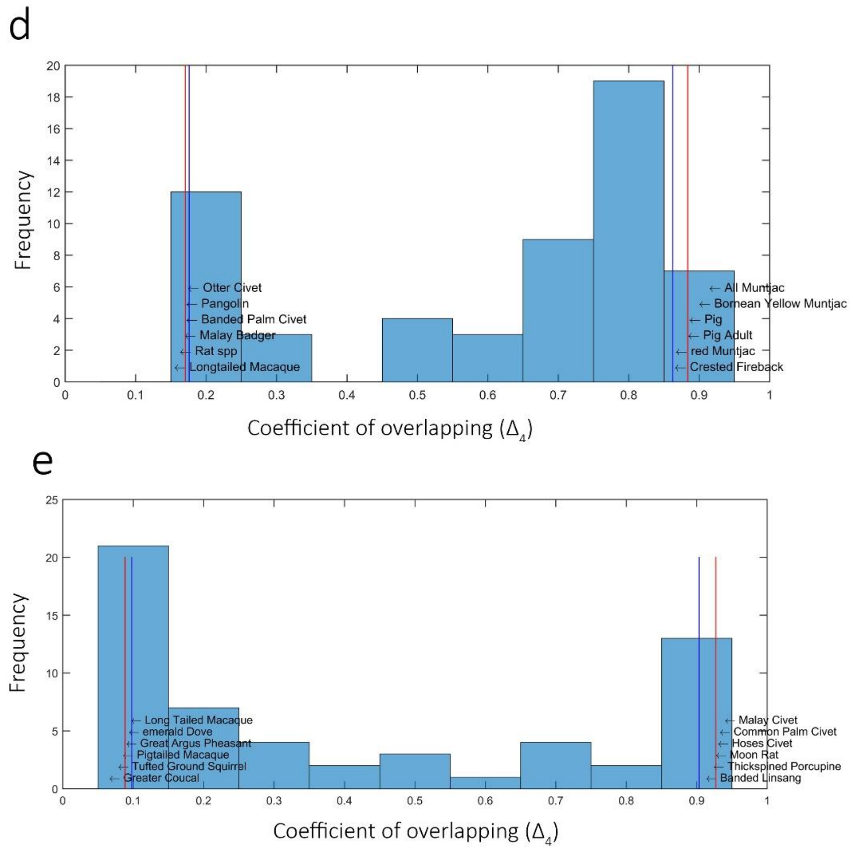

S3 Fig continued. Histogram of the frequency of coefficient of temporal overlap (Δ1 and Δ4) values between Bornean felids and all other species and species groups (n= 58. d. marbled cat; e. leopard cat. The 5th and 95th percentiles of the Δ1 and Δ4 distribution for each felid are shown in vertical red lines, and the 10th and 90th percentiles are shown in vertical blue lines.
