# Supplementary material for: Spatio-temporal ecology of sympatric felids on Borneo. Evidence for resource partitioning?
Source: PLoS One. 2018 Jul 20;13(7):e0200828. doi: 10.1371/journal.pone.0200828 (PMC6054408; doi:10.1371/journal.pone.0200828)
Supplement: S1 Table — (PDF) [file pone.0200828.s004.pdf]

Spatio-temporal ecology of sympatric felids on Borneo. Evidence for resource partitioning?

Andrew J. Hearn, Samuel A. Cushman, Joanna Ross, Benoit Goossens, Luke T.B. Hunter, and David W. Macdonald.

S1 Table. Details of the eight forest and two oil palm plantation study areas in Sabah, Malaysian Borneo. [a] Dominant land cover classes in each study area, derived from Gaveau et al. (2014), arranged in decreasing order of occurrence.

Study area	Lat/Lon	General description	Gaveau et al. (2014) landcover classes [a]
Crocker Range	5° 26’ N, 116° 02’ E	Primary, hill dipterocarp, sub-montane & montane.	Natural forest; Logged forest.
Danum Valley	4° 58’ N, 117° 46’ E	Primary, lowland & hill dipterocarp.	Natural forest; Logged forest.
Kabili-Sepilok	5° 51’ N, 117° 57’ E	Partially selectively logged, lowland Dipterocarp, heath forest & mangrove.	Natural forest; Logged forest; Intact mangrove; Non forest; Agroforest/Forest regrowth.
Kinabatangan	5° 29’ N, 118° 08’ E	Selectively logged, mosaic of forest types, including riparian forest, seasonally flooded forest, swamp forest, limestone forest.	Logged forest; Agroforest/Forest regrowth; Non forest
Malua	5° 08’ N, 117° 40’ E	Twice-logged (1960s & 2006-2007), lowland dipterocarp. High density of open logging roads and skid trails.	Logged forest; Agroforest/Forest regrowth.
Tabin	5° 14’ N, 118° 51’ E	Selectively logged (1969-1989), lowland dipterocarp. Low density of open and semi-closed logging roads.	Logged forest; Severely degraded forest.
Tawau	4° 27’ N, 117° 57’ E	Primary, lowland & hill dipterocarp, sub-montane & montane.	Natural forest, Logged forest; Agroforest/Forest regrowth
Ulu Segama	4° 59’ N, 117° 52’ E	Selectively logged (1978-1991), lowland Dipterocarp. Medium density of open and semi-closed logging roads.	Logged forest; Severely degraded forest.
Danum Palm	5° 05’ N, 117° 46’ E	Semi-mature (planted in 2000), terraced oil palm plantation. Largely open understorey. Semi-natural scrub bordering one large river and one stream.	Oil palm plantations.
Minat Teguh	5° 50’ N, 117° 53’ E	Mature (planted in 1995) oil palm plantation. Largely open understorey. Border fringed with mangrove.	Oil palm plantations; Intact mangroves; Natural forest, Non forest.
