# Supplementary material for: Spatio-temporal ecology of sympatric felids on Borneo. Evidence for resource partitioning?
Source: PLoS One. 2018 Jul 20;13(7):e0200828. doi: 10.1371/journal.pone.0200828 (PMC6054408; doi:10.1371/journal.pone.0200828)
Supplement: S2 Table — (PDF) [file pone.0200828.s005.pdf]

Spatio-temporal ecology of sympatric felids on Borneo. Evidence for resource partitioning?

Andrew J. Hearn, Samuel A. Cushman, Joanna Ross, Benoit Goossens, Luke T.B. Hunter, and David W. Macdonald·

S2 Table. Details of camera trap survey protocols for surveys of eight forest areas and two palm oil plantations in Sabah, Malaysian Borneo. [a] Camera trap grid area is defined by a 100% Minimum Convex Polygon around all camera stations. [b] We followed two survey protocols, Split-grid: where the entire grid was sequentially surveyed in two halves, and Simultaneously (Sim): where all camera stations were deployed in a single phase. [c] values within parentheses represent the number of camera stations situated along forest roads and trails, respectively.

Study area	Camera trap grid				Survey effort	
	Area (km^2) [a]	Protocol [b]	No. cam. stations [c]	Mean elevation and range (m.a.s.l)	Survey dates	No. trap days
Crocker Range	149.7	Sim.	35 (3, 32)	1029 (383-1452)	6/10/11 - 27/2/12	4059
Danum Valley (1)	66.7	Sim.	23 (2, 21)	285 (175-554)	25/10/07 - 30/12/08	3857
Danum Valley (2)	157.0	Split	79 (0, 79)	384 (153-804)	24/3/12 - 6/10/12	5837
Kabili Sepilok	49.4	Sim.	35 (0, 35)	66 (8-134)	9/2/11 - 25/5/11	2054
Kinabatangan	359.5	Split	68 (0, 68)	35 (5-135)	24/7/10 - 17/12/10	4340
Malua	102.8	Sim.	38 (38, 0)	177 (68-286)	9/7/08 - 12/2/09	3869
Tabin	71.4	Split	37 (1, 36)	140 (11-407)	16/12/09 - 22/4/10	6462
Tawau (1)	149.0	Sim.	77 (0, 77)	706 (209-1195)	21/10/12 - 30/12/13	17397
Tawau (2)	135.7	Sim.	71 (0, 71)	671 (183-1102)	6/7/13 - 11/12/14	17596
Ulu Segama	60.1	Sim.	22 (19, 3)	252 (150-408)	24/5/07 - 18/10/07	2847
Danum Palm	7.8	Sim.	23	210 (120-295)	15/3/09 - 7/7/09	2212
Minat Teguh	44.0	Sim.	33	23 (1-49)	26/5/11 - 18/8/11	1960
Totals:	1353.0	-	578	-	-	72490
