# Supplementary material for: Spatio-temporal ecology of sympatric felids on Borneo. Evidence for resource partitioning?
Source: PLoS One. 2018 Jul 20;13(7):e0200828. doi: 10.1371/journal.pone.0200828 (PMC6054408; doi:10.1371/journal.pone.0200828)
Supplement: S3 Table — (PDF) [file pone.0200828.s006.pdf]

Spatio-temporal ecology of sympatric felids on Borneo. Evidence for resource partitioning?

Andrew J. Hearn, Samuel A. Cushman, Joanna Ross, Benoit Goossens, Luke T.B. Hunter, and David W. Macdonald.

S3 Table. Covariates used in the multi-scale habitat modelling of Bornean felid's occurrence in Sabah, including variable names, metrics calculated, and data sources.

[1] Focal mean and standard deviation (SD) were calculated for all continuous variables.

Variable name	Metric[1]	Data source and decsription
Elevation	Focal mean & SD	Jarvis et al. 2008 (http://srtm.csi.cgiar.org). A 30m resolution Shuttle Radar Topographic Mission (SRTM) digital elevation model
Roughness	Focal mean & SD	A digital elevation layer (Jarvis et al. 2008) was transformed into roughness using the Geomorphometry & Gradient Metrics Toolbox (Evans et al. 2014) in ArcGIS 10.2.2 (Environmental Systems Research Incorporated, ESRI, Redlands, CA, USA, 2011).
Canopy cover	Focal mean & SD	Hansen et al. 2013 (http://earthenginepartners.appspot.com/science-2013-global-forest/download_v1.2.html). Provides % forest cover data.
Human footprint	Focal mean & SD	Wildlife Conservation (WCS) and Center for International Earth Science Information Network (CIESIN) (http://sedac.ciesin.columbia.edu/wildareas/)
Gaveau: Agroforest/forest regrowth	Focal mean	50m spatial resolution layers depicting Borneo-wide landcover with forest quality for the year 2010. Gaveau et al., (2014).
Gaveau: Logged forests	Focal mean	
Gaveau: Non forest	Focal mean	
Gaveau: Oil palm plantations	Focal mean	
Miettinen: Largescale palm production	Focal mean	A 250m spatial resolution 2010 land cover map developed by Miettinen et al. (2012)
Miettinen: Lowland forest	Focal mean	
Miettinen: Lowland mosaic	Focal mean	
Miettinen: Lowland open	Focal mean	
Miettinen: Peatswamp forest	Focal mean	
Miettinen: Plantation/regrowth	Focal mean	
SFD: Lowland Freshwater Swamp Forest	Focal mean	Unpublished historic forest layers developed by the Sabah Forestry Department, based on soil/elevation charecteristics. This layer was clipped to areas defined as forest in the Gaveau et al., 2010 dataset, to account for deforestation.
SFD: Lowland Mixed Dipterocarp & Kerangas Forest	Focal mean	
SFD: Lowland Mixed Dipterocarp Forest	Focal mean	
SFD: Lowland Mixed Dipterocarp Forest & Limestone vegetation	Focal mean	
SFD: Lowland Peat Swamp Forest	Focal mean	
SFD: Lowland Seasonal Freshwater Swamp Forest	Focal mean	
