# Supplementary material for: Spatio-temporal ecology of sympatric felids on Borneo. Evidence for resource partitioning?
Source: PLoS One. 2018 Jul 20;13(7):e0200828. doi: 10.1371/journal.pone.0200828 (PMC6054408; doi:10.1371/journal.pone.0200828)
Supplement: S4 Table — (PDF) [file pone.0200828.s007.pdf]

Spatio-temporal ecology of sympatric felids on Borneo. Evidence for resource partitioning?

Andrew J. Hearn, Samuel A. Cushman, Joanna Ross, Benoit Goossens, Luke T.B. Hunter, and David W. Macdonald

S4 Table. Table of variables used in the felid/candidate prey co-occurrence all-subsets modelling and the felid/candidate prey temporal activity overlap analysis, showing variable description, number of independent photographic records for each species/group of species ([a] 1 record/ species/ camera station/ hour) and IUCN Red List status (CR: Critically endangered; EN: Endangered; VU; Vulnerable; NT: Near threatened; LC Least concern; NA: not applicable; Red List status as of July, 2016).

Variable name	Species records included in variable	No. records [a]	IUCN Red List [b]
All mousedeer	Combination of Greater mousedeer (*Tragulus napu), Lesser mousedeer* (*T. kanchil*) records, and Mousedeer records which are unidentifiable to the species level.	7314	NA
All muntjac	Combination of Bornean Yellow Muntjac (*Muntiacus atherodes*), Southern Red Muntjac (*M. muntjak*) records, and muntjac records which are unidentifiable to the species level.	13447	NA
All partridges	Combination of Blue-breasted quail (*Synoicus chinensis*), Sabah Partridge (Arborophila graydoni), Crested partridge (*Rollulus rouloul*), Crimson-headed partridge (*Haematortyx sanguiniceps*), Red-breasted partridge (*Arborophila hyperythra*) and partridge records which are unidentifiable to the species level.	229	NA
All pheasants	Combination of Bulwer's Pheasant (*Lophura bulweri*), Crested fireback (*L. ignita*), Great Argus (*Argusianus argus*) records, and pheasant records which are unidentifiable to the species level.	4892	NA
All pittas	Combination of Bornean banded pitta (*Pitta schwaneri*), Black-headed pitta (*P. ussheri*), Blue-banded pitta (*P. arcuata*), Blue-headed pitta (*P. baudii*), Giant pitta (*P. caerulea*) and Hooded pitta (*P. sordida*) records, and pitta records which are unidentifiable to the species level.	342	NA
All small birds	Combination of all bird records, excluding pheasants, pittas and partridges.	512	NA
Banded linsang	Banded Linsang (*Prionodon linsang*)	105	LC
Banded palm civet	Banded palm civet (*Hemigalus derbyanus*)	2237	NT
Banded pitta	Bornean banded pitta (*Pitta schwaneri*)	266	LC
Bay cat	Borneo bay cat (*Catopuma badia*)	61	EN
Binturong	Binturong (Arctictis binturong)	70	VU
Blue headed pitta	Blue-headed Pitta (*Pitta baudii*)	37	VU
Bornean yellow muntjac	Bornean Yellow Muntjac (*Muntiacus atherodes*)	7119	VU
Bulwers pheasant	Bulwer's Pheasant (*Lophura bulweri*)	551	VU

S4 Table (continued).

Variable name	Species records included in variable	No. records [a]	IUCN Red List [b]
Emerald dove	Grey-capped emerald dove (C*halcophaps indica*)	363	LC
Great Argus pheasant	Great Argus (*Argusianus argus*)	2976	NT
Greater coucal	Greater Coucal (*Centropus sinensis*)	20	LC
Greater mousedeer	Greater Mousedeer (*Tragulus napu*)	1882	LC
Hoses civet	Hose's civet (*Diplogale hosei*)	281	VU
Leopard cat	Leopard cat (Prionailurus bengalensis)	1973	LC
Lesser mouse deer	Lesser mousedeer (*Tragulus kanchil*)	2638	LC
Long-tailed macaque	Long-tailed macaque (Macaca fascicularis)	946	LC
Long-tailed porcupine	Long-tailed Porcupine (*Trichys fasciculata*)	865	LC
Malay badger	Malay badger (*Mydaus javanensis*)	437	LC
Malay civet	Malay civet (*Viverra tangalunga*)	6458	LC
Malay weasel	Malay weasel (*Mustela nudipes*)	29	LC
Marbled cat	Marbled cat (Pardofelis marmorata)	205	NT
Masked palmcivet	Masked palm civet (*Paguma larvata*)	284	LC
Mongoose spp	Combination of the records of Collared mongoose (Herpestes semitorquatus) and Short-tailed mongoose (Herpestes brachyurus), and mongoose records which are unidentifiable to the species level.	272	NA
Moonrat	Moonrat (*Echinosorex gymnura*)	863	LC
Orangutan	Bornean orangutan (*Pongo pygmaeus*)	254	CR
Otter civet	Otter civet (*Cynogale bennettii*)	43	EN
Pangolin	Sunda Pangolin (*Manis javanica*)	143	CR
Pig	Bearded Pig (*Sus barbatus*) (all records)	9587	VU
Pig adult	Bearded Pig (*Sus barbatus*) (adult records only)	5977	VU
Pig juv	Bearded Pig (*Sus barbatus*) (juvenile records only)	1231	VU
Pig subadult	Bearded Pig (*Sus barbatus*) (subadult records only)	950	VU
Pig-tailed macaque	Southern pig-tailed macaque (*Macaca nemestrina*)	5219	VU
Rat spp	Combination of the records of species within the family Muridae (up to 27 species in 10 genera).	2319	NA
Red muntjac	Southern Red Muntjac (M*untiacus muntjak*)	5906	LC
Sambar deer	Sambar Deer (*Rusa unicolor*)	2751	VU
Short-tailed mongoose	Short-tailed mongoose (H*erpestes brachyurus*)	817	NT
Sun bear	Malayan Sun Bear (*Helarctos malayanus*)	914	VU
Thick-spined porcupine	Thick-spined porcupine (*Hystrix crassispinis*)	614	LC
Treeshrew spp.	Combination of the records of species within the family Tupaiidae (approx. 6 species).	587	NA
Tree squirrel	Combination of the records of several, largely arboreal squirrel species, including: Horse-tailed squirrel (*Sundasciurus hippurus*), Low's Squirrel (*S. lowii*), Prevost's squirrel (*Callosciurus prevostii*), and Thomas's flying squirrel (*Aeromys thomasi*)	364	NA
Tufted ground squirrel	Tufted ground squirrel (*Rheithrosciurus macrotis*)	327	VU
Yellow-throated marten	Yellow-throated marten (*Martes flavigula*)	312	LC
