# Supplementary material for: Spatio-temporal ecology of sympatric felids on Borneo. Evidence for resource partitioning?
Source: PLoS One. 2018 Jul 20;13(7):e0200828. doi: 10.1371/journal.pone.0200828 (PMC6054408; doi:10.1371/journal.pone.0200828)
Supplement: S5 Table — Variables showing p values <0.2 were used in the multivariate analyses. (PDF) [file pone.0200828.s008.pdf]

Spatio-temporal ecology of sympatric felids on Borneo. Evidence for resource partitioning?

Andrew J. Hearn, Samuel A. Cushman, Joanna Ross, Benoit Goossens, Luke T.B. Hunter, and David W. Macdonald.

S5 Table. Results of univariate logistic regressions to assess the relative importance of habitat variables in predicting Bornean felid occurrence, showing optimal scale of each habitat variable. Variables showing p values <0.2 were used in the multivariate analyses.

Variable	Optimal scale (m)	Coefficient	AIC	P-Value
Sunda Clouded leopard				
Elevation	120	0.00136	630.499	5.25E-05
Gaveau: Agroforest/forest regrowth	7680	-2.03649	629.089	0.00012
Gaveau: Oil palm plantations	7680	-0.45241	617.683	7.52E-07
Gaveau: Logged forests	7680	0.26937	613.737	1.07E-07
Miettinen: Lowland open	7680	-0.91899	637.082	0.00551
Miettinen: Plantation/regrowth	7680	-0.98417	620.645	2.22E-06
Miettinen: Lowland mosaic	7680	-0.72579	627.689	0.00013
SFD: Lowland Mixed Dipterocarp Forest	480	0.30057	614.076	3.72E-05
SFD: Lowland Mixed Dipterocarp Forest & Limestone vegetation	120	-0.59114	644.595	0.453
Human footprint	7680	-0.12614	610.372	1.26E-07
Roughness	7680	0.33954	627.513	2.41E-05
Canopy cover	7680	0.06582	613.916	2.98E-07
Canopy cover std	7680	-0.07639	606.944	2.24E-09
Bay cat				
Elevation	120	-73.83250	232.862	0.505
Gaveau: Agroforest/forest regrowth	480	-21.42180	227.439	0.271
Gaveau: Oil palm plantations	1920	-81.36520	222.353	0.6
Gaveau: Logged forests	240	190.14540	223.22	0.99
Miettinen: Lowland open	1920	-8233.51000	230.473	0.961
Miettinen: Plantation/regrowth	240	-1.20425	235.291	0.332
Miettinen: Lowland mosaic	1920	-139.75500	226.003	0.612
SFD: Lowland Mixed Dipterocarp Forest	1920	46.37472	221.218	0.567
SFD: Lowland Mixed Dipterocarp Forest & Limestone vegetation	7680	-3728.43000	237.053	0.989
Human footprint	3840	-0.07375	237.425	0.1197
Roughness	7680	0.25091	238.084	0.159
Canopy cover	3840	0.16767	227.957	0.02108
Canopy cover std	960	-0.58446	222.66	0.0859
Marbled cat				
Elevation	120	0.00129	475.054	0.00085
Gaveau: Agroforest/forest regrowth	7680	-2.47138	471.408	0.00073
Gaveau: Oil palm plantations	7680	-0.48467	466.581	9.87E-05
Gaveau: Logged forests	7680	0.29443	463.074	2.03E-05
Miettinen: Lowland open	7680	-1.62160	474.008	0.00839
Miettinen: Plantation/regrowth	3840	-0.59497	476.393	0.00679
Miettinen: Lowland mosaic	7680	-0.90594	471.089	0.00176

S5 Table Continued.

Variable	Optimal scale (m)	Coefficient	AIC	P-Value
Leopard cat				
Elevation	1920	-0.00117	757.009	0.00144
Gaveau: Agroforest/forest regrowth	7680	0.61839	764.908	0.08163
Gaveau: Oil palm plantations	480	0.53711	699.623	8.75E-12
Gaveau: Logged forests	480	-0.20891	725.567	3.52E-10
Miettinen: Lowland open	7680	-0.81129	755.524	0.00097
Miettinen: Plantation/regrowth	7680	-0.67206	750.488	5.86E-05
Miettinen: Lowland mosaic	3840	-0.61699	749.64	0.00013
SFD: Lowland Mixed Dipterocarp Forest	240	-0.14167	741.72	4.62E-07
SFD: Lowland Mixed Dipterocarp Forest & Limestone vegetation	7680	-6.32376	757.811	0.0386
Human footprint	960	0.01841	766.399	0.21537
Roughness	3840	-0.11632	764.172	0.0537
Canopy cover	120	-0.03296	745.432	9.72E-06
Canopy cover std	240	0.17063	751.814	9.58E-05
