# Supplementary material for: Spatio-temporal ecology of sympatric felids on Borneo. Evidence for resource partitioning?
Source: PLoS One. 2018 Jul 20;13(7):e0200828. doi: 10.1371/journal.pone.0200828 (PMC6054408; doi:10.1371/journal.pone.0200828)
Supplement: S6 Table — The coefficients of overlap (Δ1 and Δ4) are accompanied by the upper and lower values of the 95% confidence limits. (PDF) [file pone.0200828.s009.pdf]

Spatio-temporal ecology of sympatric felids on Borneo. Evidence for resource partitioning?

S6 Table. Table of Overlaps of temporal activity patterns between Bornean wild cat species pairs and between Bornean wild cats and their potential prey species, as estimated by kernel density estimates. The coefficients of overlap (Δ_1 and Δ_4) are accompanied by the upper and lower values of the 95 % confidence limits.

	Clouded leopard			Clouded leopard Males			Clouded leopard Females			Bay cat			Marbled cat			Leopard cat		
	Δ_4	lower	upper	Δ_4	lower	upper	Δ_4	lower	upper	Δ_1	lower	upper	Δ_4	lower	upper	Δ_4	lower	upper
Felids:																		
Clouded leopard	-	-	-	-	-	-	-	-	-	0.398	0.331	0.468	0.521	0.463	0.576	0.742	0.707	0.777
Clouded leopard male	-	-	-	-	-	-	0.873	0.789	0.946	0.396	0.327	0.468	0.520	0.459	0.579	0.739	0.701	0.779
Clouded leopard female	-	-	-	0.873	0.789	0.946				0.411	0.307	0.519	0.525	0.413	0.626	0.734	0.646	0.825
Bay cat	0.398	0.331	0.468	0.396	0.327	0.468	0.411	0.307	0.519				0.787	0.695	0.871	0.147	0.091	0.209
Marbled cat	0.521	0.463	0.576	0.520	0.459	0.579	0.525	0.413	0.626	0.787	0.695	0.871				0.264	0.206	0.305
Leopard cat	0.742	0.707	0.777	0.739	0.701	0.779	0.734	0.646	0.825	0.147	0.091	0.209	0.264	0.206	0.305	-	-	-
Potential prey:																		
All mousedeer	0.803	0.774	0.832	0.824	0.786	0.847	0.759	0.686	0.830	0.428	0.356	0.504	0.609	0.551	0.664	0.581	0.561	0.600
All muntjac	0.518	0.483	0.553	0.529	0.492	0.564	0.531	0.435	0.618	0.802	0.733	0.870	0.914	0.878	0.945	0.261	0.248	0.274
All partridges	0.378	0.338	0.420	0.379	0.336	0.421	0.383	0.280	0.484	0.761	0.669	0.859	0.769	0.702	0.838	0.109	0.082	0.136
All pheasants	0.372	0.339	0.405	0.373	0.336	0.408	0.380	0.280	0.473	0.861	0.790	0.933	0.843	0.792	0.889	0.104	0.090	0.117
All pittas	0.390	0.348	0.430	0.391	0.349	0.431	0.392	0.294	0.490	0.853	0.766	0.930	0.821	0.763	0.877	0.120	0.097	0.145
All small birds	0.395	0.356	0.433	0.396	0.357	0.434	0.399	0.295	0.501	0.898	0.812	0.968	0.848	0.788	0.902	0.123	0.102	0.144
Banded linsang	0.704	0.640	0.768	0.700	0.638	0.765	0.706	0.595	0.807	0.118	0.048	0.196	0.209	0.139	0.280	0.913	0.852	0.962
Banded palm civet	0.629	0.593	0.664	0.624	0.527	0.727	0.653	0.556	0.755	0.082	0.034	0.141	0.171	0.125	0.218	0.859	0.836	0.881
Banded pitta	0.367	0.324	0.410	0.367	0.325	0.412	0.372	0.274	0.475	0.853	0.767	0.932	0.796	0.737	0.853	0.100	0.075	0.125
Binturong	0.716	0.612	0.815	0.703	0.596	0.806	0.719	0.581	0.839	0.625	0.506	0.731	0.674	0.580	0.762	0.498	0.388	0.604
Blue-headed pitta	0.411	0.334	0.494	0.412	0.332	0.489	0.405	0.293	0.526	0.880	0.738	0.970	0.809	0.702	0.906	0.151	0.086	0.221
Bornean yellow muntjac	0.462	0.411	0.478	0.445	0.408	0.480	0.447	0.350	0.543	0.443	0.351	0.537	0.899	0.856	0.934	0.175	0.161	0.189
Bulwers pheasant	0.393	0.356	0.431	0.394	0.354	0.432	0.399	0.298	0.497	0.791	0.711	0.876	0.835	0.774	0.888	0.124	0.104	0.144
Chestnut necklaced partridge	0.409	0.339	0.484	0.410	0.330	0.489	0.410	0.293	0.524	0.792	0.653	0.914	0.782	0.659	0.894	0.135	0.074	0.200
Collared mongoose	0.371	0.323	0.420	0.372	0.323	0.425	0.368	0.261	0.468	0.833	0.719	0.927	0.762	0.688	0.831	0.112	0.080	0.148
Common palm civet	0.691	0.651	0.731	0.688	0.643	0.730	0.700	0.600	0.793	0.091	0.051	0.175	0.211	0.159	0.263	0.932	0.901	0.959
Common porcupine	0.632	0.593	0.670	0.627	0.589	0.665	0.659	0.560	0.761	0.071	0.033	0.145	0.177	0.133	0.226	0.852	0.828	0.876
Crested fireback	0.397	0.364	0.433	0.399	0.362	0.436	0.400	0.300	0.502	0.800	0.726	0.879	0.864	0.816	0.908	0.127	0.111	0.144
Crested partridge	0.380	0.336	0.425	0.381	0.334	0.431	0.384	0.286	0.485	0.761	0.662	0.860	0.763	0.682	0.841	0.111	0.080	0.143
Elephant	0.657	0.606	0.709	0.644	0.590	0.692	0.704	0.612	0.795	0.610	0.521	0.698	0.725	0.662	0.789	0.479	0.439	0.521

S6 Table. (Continued).

	Clouded leopard			Clouded leopard Males			Clouded leopard Females			Bay cat			Marbled cat			Leopard cat		
	Dhat4	lower	upper	Dhat4	lower	upper	Dhat4	lower	upper	Dhat1	lower	upper	Dhat4	lower	upper	Dhat4	lower	upper
Potential prey:																		
Greater coucal	0.306	0.212	0.413	0.303	0.203	0.407	0.327	0.209	0.453	0.839	0.660	0.963	0.685	0.535	0.818	0.065	0.003	0.155
Greater mousedeer	0.869	0.832	0.902	0.860	0.819	0.897	0.854	0.777	0.920	0.305	0.243	0.376	0.457	0.400	0.509	0.795	0.770	0.820
Hoses civet	0.723	0.675	0.768	0.719	0.662	0.769	0.728	0.628	0.824	0.138	0.078	0.204	0.233	0.179	0.292	0.930	0.891	0.964
Lesser mousedeer	0.670	0.635	0.707	0.672	0.634	0.711	0.645	0.562	0.727	0.519	0.438	0.600	0.704	0.648	0.754	0.423	0.399	0.445
Long-tailed macaque	0.353	0.317	0.387	0.351	0.313	0.389	0.365	0.269	0.465	0.850	0.754	0.932	0.693	0.622	0.758	0.096	0.080	0.112
Long-tailed porcupine	0.633	0.598	0.667	0.630	0.591	0.672	0.645	0.549	0.738	0.071	0.020	0.127	0.154	0.109	0.202	0.867	0.837	0.894
Malay badger	0.670	0.630	0.711	0.668	0.625	0.710	0.663	0.568	0.753	0.084	0.032	0.144	0.168	0.118	0.224	0.897	0.862	0.927
Malay civet	0.734	0.703	0.766	0.732	0.699	0.767	0.731	0.645	0.814	0.132	0.074	0.193	0.254	0.208	0.308	0.939	0.920	0.959
Malay weasel	0.396	0.308	0.492	0.396	0.305	0.491	0.405	0.278	0.533	0.777	0.614	0.916	0.770	0.625	0.898	0.140	0.058	0.238
Masked palm civet	0.785	0.739	0.830	0.781	0.733	0.830	0.775	0.686	0.858	0.195	0.125	0.274	0.331	0.272	0.394	0.893	0.846	0.934
Mongoose spp	0.371	0.328	0.415	0.370	0.326	0.419	0.374	0.276	0.471	0.878	0.787	0.939	0.749	0.687	0.815	0.106	0.082	0.134
Moonrat	0.675	0.639	0.712	0.672	0.632	0.712	0.679	0.585	0.772	0.092	0.037	0.150	0.187	0.139	0.240	0.926	0.900	0.951
Orangutan	0.366	0.322	0.408	0.363	0.318	0.409	0.381	0.285	0.480	0.880	0.786	0.953	0.712	0.640	0.780	0.112	0.083	0.142
Otter civet	0.648	0.563	0.738	0.644	0.564	0.729	0.668	0.564	0.776	0.097	0.020	0.178	0.173	0.095	0.262	0.885	0.787	0.956
Pangolin	0.643	0.578	0.702	0.639	0.572	0.706	0.616	0.507	0.714	0.092	0.031	0.161	0.171	0.110	0.230	0.816	0.759	0.865
Pig	0.611	0.577	0.644	0.611	0.573	0.644	0.612	0.513	0.712	0.763	0.685	0.832	0.885	0.839	0.928	0.349	0.333	0.366
Pig adult	0.595	0.562	0.629	0.594	0.558	0.630	0.598	0.509	0.692	0.787	0.718	0.861	0.883	0.830	0.928	0.334	0.318	0.352
Pig juv	0.450	0.415	0.485	0.448	0.409	0.490	0.460	0.362	0.570	0.895	0.819	0.949	0.818	0.754	0.876	0.186	0.165	0.208
Pig subadult	0.477	0.438	0.515	0.474	0.435	0.514	0.492	0.391	0.586	0.863	0.789	0.921	0.861	0.804	0.914	0.220	0.195	0.244
Pig-tailed macaque	0.332	0.298	0.364	0.331	0.296	0.365	0.343	0.258	0.438	0.899	0.832	0.959	0.712	0.659	0.771	0.085	0.073	0.097
Rat spp	0.635	0.602	0.670	0.632	0.595	0.667	0.649	0.548	0.748	0.083	0.037	0.135	0.162	0.119	0.207	0.874	0.853	0.895
Red muntjac	0.621	0.586	0.658	0.622	0.585	0.659	0.624	0.527	0.718	0.747	0.679	0.806	0.866	0.821	0.905	0.356	0.338	0.373
Sambar deer	0.905	0.874	0.937	0.892	0.856	0.926	0.853	0.770	0.922	0.419	0.359	0.493	0.570	0.525	0.618	0.700	0.677	0.721
Short-tailed mongoose	0.358	0.320	0.395	0.357	0.322	0.394	0.366	0.273	0.461	0.917	0.836	0.976	0.751	0.691	0.812	0.099	0.081	0.116
Sun bear	0.723	0.679	0.765	0.711	0.665	0.755	0.726	0.629	0.814	0.601	0.521	0.682	0.766	0.705	0.823	0.497	0.466	0.529
Thick-spinedporcupine	0.680	0.640	0.720	0.677	0.636	0.720	0.681	0.584	0.769	0.096	0.039	0.150	0.189	0.141	0.241	0.923	0.891	0.951
Tree shrew spp.	0.422	0.385	0.460	0.423	0.386	0.462	0.423	0.326	0.526	0.747	0.663	0.835	0.815	0.756	0.873	0.156	0.135	0.179
Tree squirrel	0.425	0.383	0.466	0.427	0.385	0.469	0.428	0.330	0.528	0.750	0.659	0.838	0.837	0.773	0.895	0.159	0.133	0.187
Tufted ground squirrel	0.315	0.274	0.356	0.314	0.272	0.357	0.323	0.234	0.421	0.782	0.688	0.872	0.629	0.555	0.699	0.078	0.056	0.102
Yellow throated marten	0.402	0.356	0.447	0.410	0.364	0.458	0.433	0.333	0.537	0.852	0.743	0.939	0.810	0.738	0.876	0.159	0.128	0.191
